# Carboxylic acid reductase-dependent biosynthesis of eugenol and related allylphenols

**DOI:** 10.1186/s12934-023-02246-4

**Published:** 2023-11-18

**Authors:** Erik K. R. Hanko, Kris Niño G. Valdehuesa, Koen J. A. Verhagen, Jakub Chromy, Ruth A. Stoney, Jeremy Chua, Cunyu Yan, Johannes A. Roubos, Joep Schmitz, Rainer Breitling

**Affiliations:** 1 Manchester Institute of Biotechnology, Faculty of Science and Engineering, University of Manchester, 131 Princess Street, Manchester, M1 7DN UK; 2 dsm-firmenich, Science & Research, P.O. Box 1, Delft, 2600 MA The Netherlands

**Keywords:** Carboxylic acid reductase, Phenylpropanoid, Monolignol, Allylphenol, Eugenol, Bioconversion, *Escherichia coli*

## Abstract

**Background:**

(Hydroxy)cinnamyl alcohols and allylphenols, including coniferyl alcohol and eugenol, are naturally occurring aromatic compounds widely utilised in pharmaceuticals, flavours, and fragrances. Traditionally, the heterologous biosynthesis of (hydroxy)cinnamyl alcohols from (hydroxy)cinnamic acids involved CoA-dependent activation of the substrate. However, a recently explored alternative pathway involving carboxylic acid reductase (CAR) has proven efficient in generating the (hydroxy)cinnamyl aldehyde intermediate without the need for CoA activation. In this study, we investigated the application of the CAR pathway for whole-cell bioconversion of a range of (hydroxy)cinnamic acids into their corresponding (hydroxy)cinnamyl alcohols. Furthermore, we sought to extend the pathway to enable the production of a variety of allylphenols and allylbenzene.

**Results:**

By screening the activity of several heterologously expressed enzymes in crude cell lysates, we identified the combination of *Segniliparus rugosus* CAR (SrCAR) and *Medicago sativa* cinnamyl alcohol dehydrogenase (MsCAD2) as the most efficient enzymatic cascade for the two-step reduction of ferulic acid to coniferyl alcohol. To optimise the whole-cell bioconversion in *Escherichia coli*, we implemented a combinatorial approach to balance the gene expression levels of SrCAR and MsCAD2. This optimisation resulted in a coniferyl alcohol yield of almost 100%. Furthermore, we extended the pathway by incorporating coniferyl alcohol acyltransferase and eugenol synthase, which allowed for the production of eugenol with a titre of up to 1.61 mM (264 mg/L) from 3 mM ferulic acid. This improvement in titre surpasses previous achievements in the field employing a CoA-dependent coniferyl alcohol biosynthesis pathway. Our study not only demonstrated the successful utilisation of the CAR pathway for the biosynthesis of diverse (hydroxy)cinnamyl alcohols, such as *p*-coumaryl alcohol, caffeyl alcohol, cinnamyl alcohol, and sinapyl alcohol, from their corresponding (hydroxy)cinnamic acid precursors but also extended the pathway to produce allylphenols, including chavicol, hydroxychavicol, and methoxyeugenol. Notably, the microbial production of methoxyeugenol from sinapic acid represents a novel achievement.

**Conclusion:**

The combination of SrCAR and MsCAD2 enzymes offers an efficient enzymatic cascade for the production of a wide array of (hydroxy)cinnamyl alcohols and, ultimately, allylphenols from their respective (hydroxy)cinnamic acids. This expands the range of value-added molecules that can be generated using microbial cell factories and creates new possibilities for applications in industries such as pharmaceuticals, flavours, and fragrances. These findings underscore the versatility of the CAR pathway, emphasising its potential in various biotechnological applications.

**Supplementary Information:**

The online version contains supplementary material available at 10.1186/s12934-023-02246-4.

## Background

(Hydroxy)cinnamyl alcohols and allylphenols are two closely related groups of aromatic compounds commonly found in plants. The hydroxycinnamyl alcohols, namely coniferyl alcohol, *p*-coumaryl alcohol, and sinapyl alcohol, are synthesised through the phenylpropanoid pathway from the aromatic amino acids phenylalanine or tyrosine [1]. These three hydroxycinnamyl alcohols, also known as monolignols, play a crucial role in plant development by serving as fundamental building blocks for the biosynthesis of lignin and lignans. Lignin is essential for the formation and structural integrity of plant cell walls, while lignans are involved in various plant functions, including defence mechanisms and regulation of growth processes [2]. Moreover, in addition to their pivotal role in plant physiology, the monolignols coniferyl alcohol and *p*-coumaryl alcohol act as precursors for the synthesis of the allylphenols eugenol and chavicol, respectively. These allylphenols are important constituents of plant essential oils, contributing to the characteristic taste and aroma of numerous fruits, herbs, and spices [3].

**Fig. 1:**
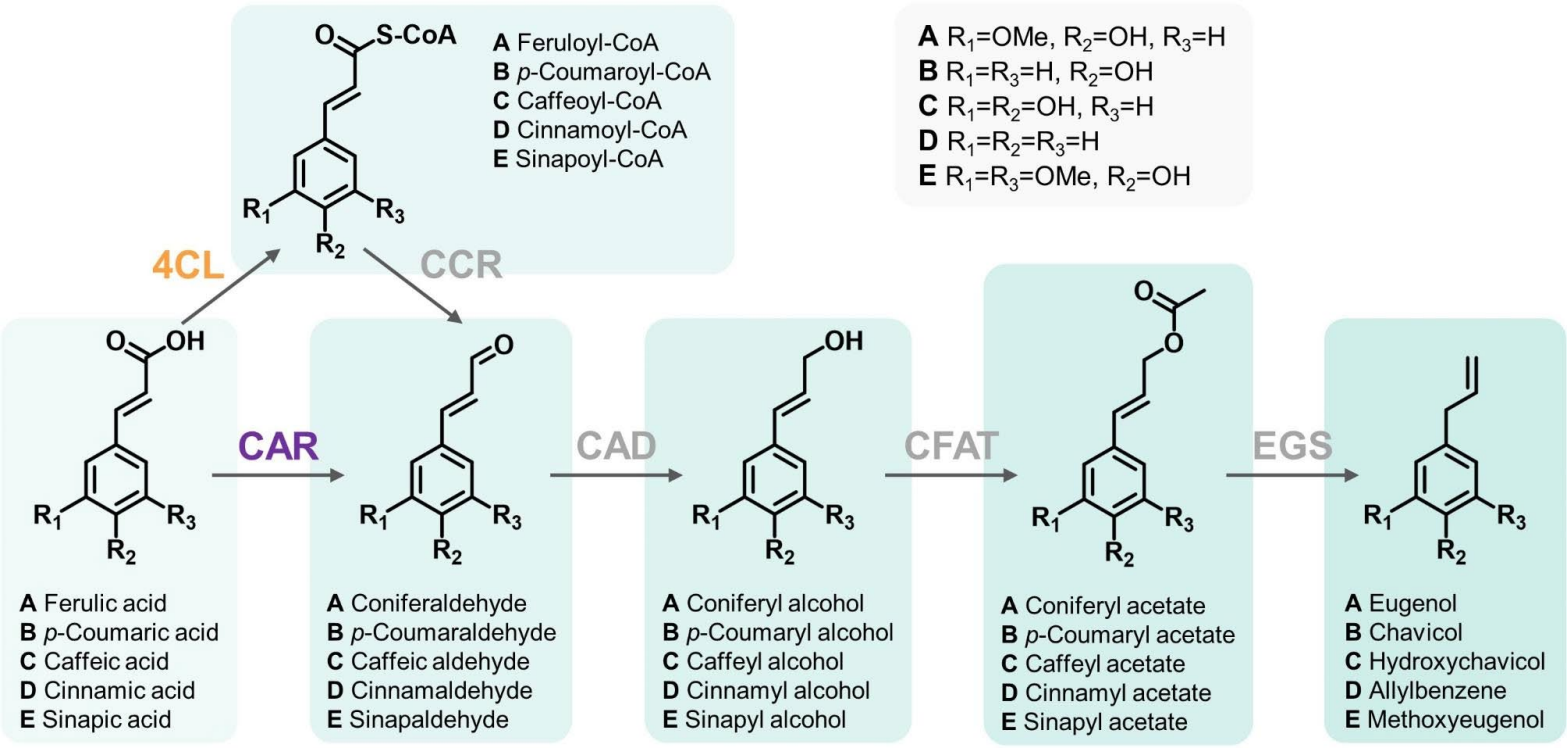
Pathway for the biosynthesis of allylphenols and allylbenzene involving CoA-dependent activation (orange) or carboxylic acid reductase-mediated reduction (purple) of the (hydroxy)cinnamic acid substrate. Enzyme abbreviations: 4CL, 4-coumarate-CoA ligase; CCR, cinnamyl CoA reductase; CAR, carboxylic acid reductase; CAD, cinnamyl alcohol dehydrogenase; CFAT, coniferyl alcohol acyltransferase; EGS, eugenol synthase

The potential applications of (hydroxy)cinnamyl alcohols and allylphenols in the flavour and fragrance industries [4], as well as their suitability as chemical building blocks for the manufacture of biopolymers [5–7], have generated increasing interest in their microbial biosynthesis. To produce (hydroxy)cinnamyl alcohols from their respective (hydroxy)cinnamic acid precursors in a microbial host, most studies have adopted the phenylpropanoid pathway commonly found in plants. This pathway involves a series of enzymatic reactions [1]. Firstly, the (hydroxy)cinnamic acid substrate is activated through the action of 4-coumarate-CoA ligase. This activation results in the formation of (hydroxy)cinnamoyl-CoA, which serves as an intermediate (Fig. 1). Subsequently, cinnamyl CoA reductase catalyses the reduction of (hydroxy)cinnamoyl-CoA, leading to the formation of (hydroxy)cinnamyl aldehyde. To complete the synthesis of (hydroxy)cinnamyl alcohols, the aldehyde is further reduced to the desired alcohol form. This reduction step can be facilitated either by endogenous alcohol dehydrogenases or by introducing heterologously expressed alcohol dehydrogenases into the microbial host [8–13]. The achieved yields of coniferyl alcohol through supplementation with ferulic acid using the CoA-dependent pathway in *Escherichia coli* show a considerable range, varying from 4–91% [8–11, 13]. Recently, an alternative pathway that enables the direct reduction of (hydroxy)cinnamic acids to their corresponding (hydroxy)cinnamyl aldehydes has been investigated [14, 15]. This pathway involves the use of carboxylic acid reductase (CAR), eliminating the need for CoA-dependent activation of the substrate (Fig. 1). By employing an enzymatic cascade consisting of *Nocardia iowensis* CAR and *Coptotermes gestroi* aldo-keto reductase, a coniferyl alcohol yield of 97% was achieved under optimised growth conditions [15].

Allylphenols can be produced from hydroxycinnamyl alcohols through two enzymatic steps (Fig. 1). Firstly, the hydroxycinnamyl alcohol is acetylated by an acyltransferase [16]. Subsequently, the resulting hydroxycinnamyl acetate is reduced to form the allylphenol [17]. Robinson et al. employed a pathway comprising *Petunia hybrida* coniferyl alcohol acyltransferase (PhCFAT) and *Ocimum basilicum* eugenol synthase (ObEGS) in combination with a CoA-dependent hydroxycinnamyl alcohol biosynthesis pathway to produce eugenol and chavicol from ferulic acid and *p*-coumaric acid, respectively [10]. However, the obtained titres of eugenol and chavicol were relatively low, with 102 mg/L and 28 mg/L, respectively, and a notable amount of substrate remained unconverted [10]. Moreover, the CoA-dependent pathway developed by Chen et al. [8] was recently employed in conjunction with PhCFAT and *Petunia hybrida* EGS in a bacterial co-culture system, resulting in the production of up to 244 mg/L eugenol from ferulic acid [13]. Other studies have reported the biosynthesis of allylphenols in recombinant strawberries by overexpressing ObEGS [18], or in *E. coli* expressing cinnamyl alcohol acyltransferase and propenylphenol synthase from *Larrea tridentata* and supplementing the bacterial culture with the respective hydroxycinnamyl alcohol precursors [19].

In this study, we demonstrate the successful conversion of hydroxycinnamic acids into allylphenols using a CAR enzyme-mediated whole-cell bioconversion approach. First, we identify *Segniliparus rugosus* CAR and *Medicago sativa* cinnamyl alcohol dehydrogenase as the best-performing enzyme candidates in vitro. Next, we optimise their expression levels through a combinatorial approach. This enabled the efficient in vivo biosynthesis of various (hydroxy)cinnamyl alcohols, achieving a nearly complete conversion of ferulic acid into coniferyl alcohol. Subsequently, by extending the pathway using PhCFAT and ObEGS, we demonstrate the production of eugenol, chavicol, hydroxychavicol, and methoxyeugenol from their respective hydroxycinnamic acid precursors in *E. coli* (Fig. 1). The obtained titres range from 0.15 to 0.26 g/L. These findings highlight the feasibility of using a promiscuous CAR-based pathway for microbial production of allylphenols as a viable alternative to plant extraction methods.

## Results and discussion

### Selection of enzymes

To establish a biosynthesis pathway that converts ferulic acid into eugenol without the need for CoA-dependent activation of the substrate, we first sought to identify the optimum combination of enzymes capable of catalysing the double reduction of ferulic acid into coniferyl alcohol (Fig. 1). For this purpose, we performed enzyme activity screening assays using two carboxylic acid reductases (CAR), two alcohol dehydrogenases, and one aldo-keto reductase (AKR).

In a recent study by Tramontina et al., the authors evaluated five CARs for their ability to reduce ferulic acid, and identified the CARs from *Nocardia iowensis* (NiCAR) and *Segniliparus rugosus* (SrCAR) as the top-performing enzyme candidates [15]. In their work, NiCAR was ultimately selected, in conjunction with *Coptotermes gestroi* AKR-1 (CgAKR-1), to catalyse both the in vitro and in vivo conversion of ferulic acid into coniferyl alcohol [15]. In this study, along with CgAKR-1, we included *Pseudomonas* sp. strain HR199 coniferyl alcohol dehydrogenase (PsCalA) and *Medicago sativa* cinnamyl alcohol dehydrogenase (MsCAD2) for activity screening. These enzymes have previously been shown to effectively reduce various (hydroxy)cinnamyl aldehydes to their corresponding alcohols [10, 20].

First, we monitored the conversion of ferulic acid to coniferyl aldehyde using whole-cell lysates of *Escherichia coli* NEB5α expressing either NiCAR or SrCAR. The CARs were each coexpressed with *Bacillus subtilis* 4’-phosphopantetheinyl transferase (Sfp), which is essential for CAR activation [21]. Among the two tested enzymes, SrCAR exhibited higher activity than NiCAR, resulting in a 28.2% product yield after a 3-hour incubation period (Table 1). This finding contrasts with the observations of Tramontina et al., who reported a higher yield for NiCAR in their assays [15]. It is important to note, however, that their assays employed purified enzymes rather than whole-cell lysates for activity screening. In addition to coniferyl aldehyde we observed low levels of coniferyl alcohol production, which can be attributed to endogenous aldo-keto reductases and alcohol dehydrogenases present in the *E. coli* NEB5α whole-cell lysate [22]. Among the three enzymes tested for the conversion of coniferyl aldehyde to coniferyl alcohol, MsCAD2 exhibited the best performance with a product yield of 19.3%, followed by PsCAD and CgAKR1 with product yields of 14.5% and 4.5%, respectively.

Next, we investigated the double reduction of ferulic acid to coniferyl alcohol by combining the cell lysates containing the individual enzymes. The most efficient two-step reaction was achieved using SrCAR in conjunction with MsCAD2, resulting in a product yield of 50.3% coniferyl alcohol (Table 1). The second-best conversion was obtained with the enzymatic cascade composed of NiCAR and MsCAD2, yielding 33.6% coniferyl alcohol. These results are consistent with the previous observation that NiCAR and SrCAR performed similarly well in in vitro screens using purified enzymes as catalysts [15]. Based on their performance in the activity screening using whole-cell lysates, SrCAR and MsCAD2 were selected to be evaluated for the in vivo conversion of ferulic acid into coniferyl alcohol.

**Table 1:** Activity screening of whole-cell lysates of *E. coli* NEB5α expressing the individual enzymes for the two-step conversion of ferulic acid into coniferyl alcohol via coniferyl aldehyde

Enzyme	Substrate	Product yield	
		Coniferyl aldehyde (%)	Coniferyl alcohol (%)
NiCAR	Ferulic acid	4.6	0.02
SrCAR	Ferulic acid	28.2	0.26
PsCAD	Coniferyl aldehyde	-	14.5
MsCAD2	Coniferyl aldehyde	-	19.3
CgAKR1	Coniferyl aldehyde	-	4.5
NiCAR + PsCAD	Ferulic acid	3.3	17.9
NiCAR + MsCAD2	Ferulic acid	1.1	33.6
NiCAR + CgAKR1	Ferulic acid	2.3	13.3
SrCAR + PsCAD	Ferulic acid	12.1	26.0
SrCAR + MsCAD2	Ferulic acid	6.3	50.3
SrCAR + CgAKR1	Ferulic acid	20.2	8.4

### Pathway optimisation for the in vivo bioconversion of ferulic acid into coniferyl alcohol

**Fig. 2:**
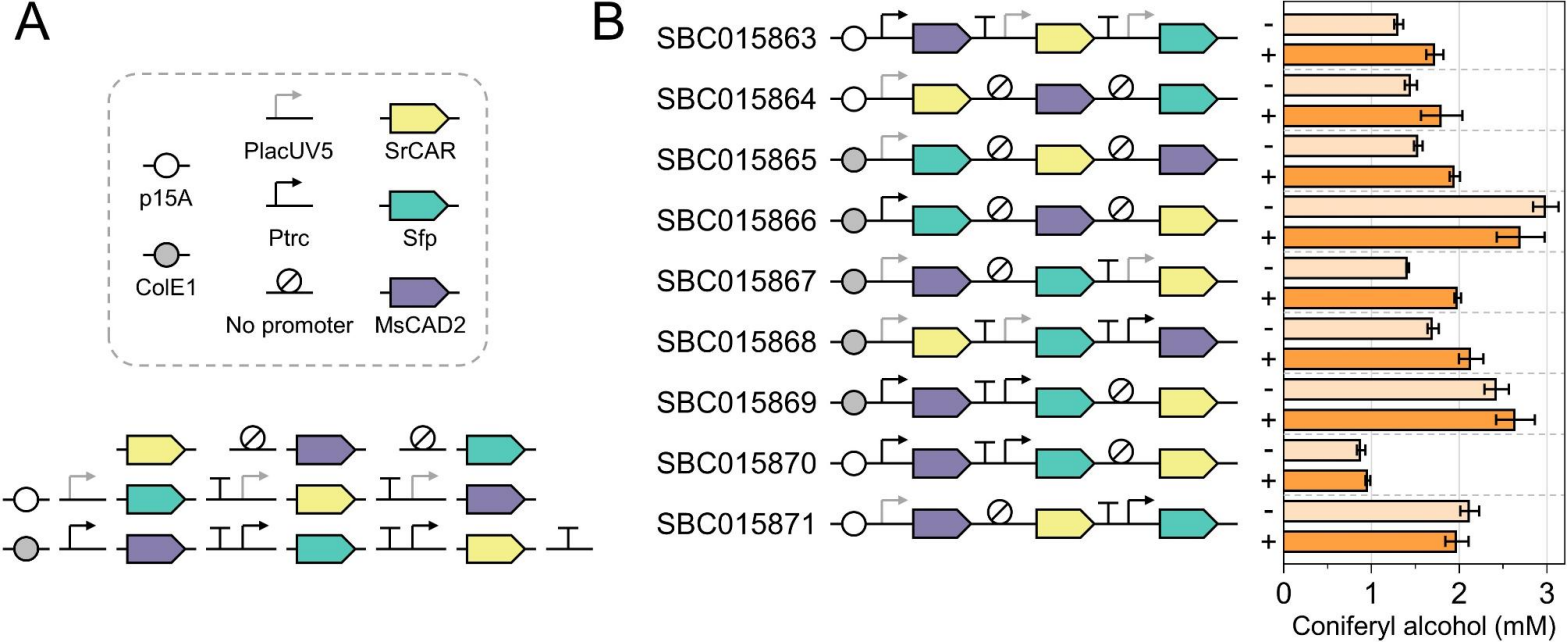
Combinatorial optimisation of the coniferyl alcohol biosynthesis pathway. **A** The pathway converting ferulic acid into coniferyl alcohol involves three genes – SrCAR, Sfp, and MsCAD2. A combinatorial library of plasmids was designed to encode this pathway, with variations in the origin of replication, order of pathway genes, and promoter parts. **B** Pathway variants that were tested for the in vivo production of coniferyl alcohol. *E. coli* NEB5α carrying the individual plasmids were grown at 30℃ for 20 h in TBP medium supplemented with 0.4% glycerol and 3 mM ferulic acid. Error bars represent standard deviations of three biological replicates. Cells were grown in the absence (−) and presence (+) of IPTG

After selecting the appropriate enzymes, we designed and constructed the biosynthesis pathway for the whole-cell in vivo bioconversion of ferulic acid into coniferyl alcohol in *E. coli* NEB5α. To identify the optimum balance of gene expression levels, we employed a combinatorial pathway design approach, which has been successfully utilised in the microbial production of fine chemicals [23]. Three parameters were taken into consideration for controlling the expression level of each pathway gene: the strength of its promoter, its position within the pathway, and the plasmid origin of replication (Fig. 2A). To create the combinatorial library of plasmids we employed two IPTG-inducible, varying-strength promoters (P_*trc*_ and P_*lacUV5*_), as well as two different origins of replication (medium copy number, p15A; and high copy number, ColE1) [24]. Intergenic regions may contain any of the two promoters or none at all, and genes may occupy any position in the pathway. Using three genes in total – SrCAR, Sfp, and MsCAD2 – this can result in 216 possible combinations. A design of experiments (DoE) approach was employed to statistically reduce this large number of potential pathway designs to nine representative ones, covering the entire design space (Fig. 2B) [23]. These nine pathway variants were assembled and introduced into *E. coli* NEB5α. Subsequently, the cells were supplemented with ferulic acid and the biosynthesis of coniferyl aldehyde and coniferyl alcohol was monitored for 48 h. Among the tested pathways, the top-performing design (SBC015866) yielded 2.99 mM coniferyl alcohol (538 mg/L) from 3 mM ferulic acid within 20 h (Fig. 2B). This titre demonstrates nearly complete conversion of the substrate into the desired product. The second-best pathway, SBC015869, produced 2.64 mM coniferyl alcohol. Notably, the difference in product titres between uninduced and induced cultures was minimal, with a maximum percentage difference of 34% (SBC015867). This suggests not only a considerable level of leaky expression of the inducible promoters, but also that gene expression levels were already reasonably well-balanced even without the addition of the inducer IPTG. Coniferyl aldehyde was detected in all nine pathway variants, but only at levels below 1% of the coniferyl alcohol titre, indicating efficient flux through the two-step pathway.

It should be noted that coniferyl alcohol titres decreased by up to 50% after reaching their maximum production at 20–24 h (Additional file 1: Figure S1). This drop in titre has been observed previously in *E. coli* for both coniferyl alcohol and caffeyl alcohol and was hypothesised to be due to either product consumption or volatility [8, 9]. To determine the cause, we monitored the titre of coniferyl alcohol over a 24-hour period when added to the culture medium in the presence and absence of *E. coli* NEB5α cells. In culture medium without cells, the level of coniferyl alcohol remained constant, while in cultures containing *E. coli* NEB5α, the titre decreased over time (Additional file 1: Figure S2). This finding suggests that the decrease in coniferyl alcohol titre is the result of cell metabolism rather than volatility. In fact, we observed the accumulation of coniferyl aldehyde in cultures containing *E. coli* NEB5α, indicating the oxidation of coniferyl alcohol likely catalysed by endogenous alcohol dehydrogenases.

Because SBC015866 showed a nearly complete in vivo conversion of ferulic acid into coniferyl alcohol, no further pathway optimisation was attempted. The yield that was achieved using an enzymatic cascade composed of SrCAR and MsCAD2, and after optimisation of gene expression levels, is comparable to the yield of 97% that was previously obtained using NiCAR in conjunction with CgAKR1 [15]. However, it surpasses the yields that have been reported for the bioconversion of ferulic acid into coniferyl alcohol via feruloyl-CoA, ranging from 4–91% [8–11, 13].

### Production of eugenol by pathway extension

Eugenol is a volatile aromatic compound that is utilised extensively in the flavour and fragrance industries due to its spicy and clove-like aroma [25]. It can be obtained in two steps through acetylation of coniferyl alcohol, followed by the reduction of coniferyl acetate to form eugenol (Fig. 1). An optimised pathway for the production of eugenol from coniferyl alcohol has been previously developed (SBC009876) [10]. It comprises *Petunia hybrida* coniferyl alcohol acyltransferase (PhCFAT) and *Ocimum basilicum* eugenol synthase (ObEGS). *E. coli* DH5α Δ*tyrR* Δ*pheLA* carrying this pathway produced 0.17 mM eugenol when supplemented with 3 mM coniferyl alcohol [10].

**Fig. 3:**
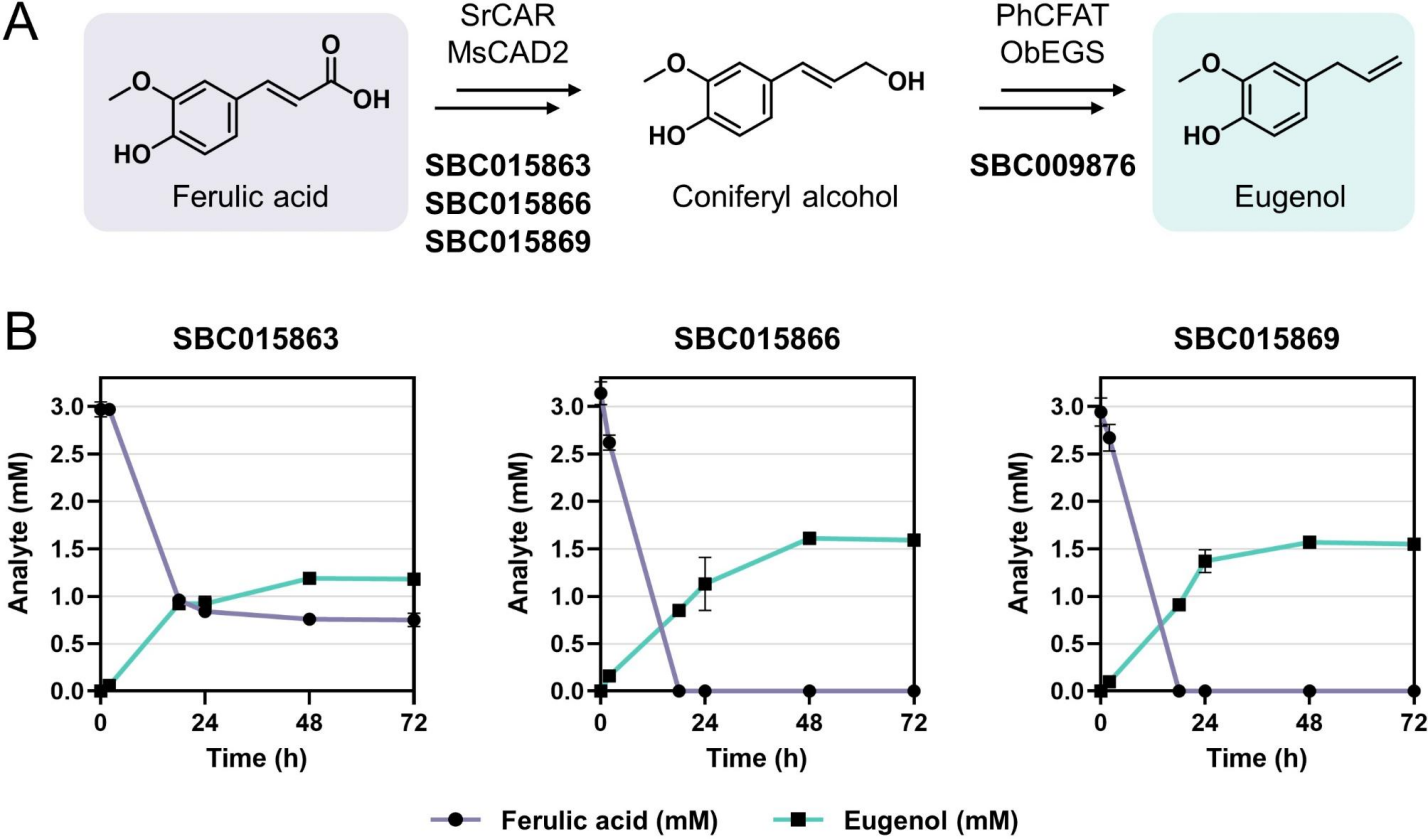
Bioconversion of ferulic acid into eugenol using the CAR-dependent pathway. **A** The eugenol biosynthesis pathway is split into two modules. The first module consists of SrCAR and MsCAD2 enzymes, catalysing the two-step reduction of ferulic acid, resulting in the formation of coniferyl alcohol. The second module comprises PhCFAT and ObEGS enzymes, catalysing the two-step conversion of coniferyl alcohol into eugenol. **B** Production of eugenol in *E. coli* NEB5α carrying SBC009876 in combination with one of the three indicated plasmids. At time point zero, cells were supplemented with 3 mM ferulic acid, and expression of pathway enzymes was induced by addition of IPTG. Error bars represent standard deviations of biological triplicates

Given the excellent conversion rate that was achieved in our combinatorial plasmid library screen, we sought to combine both pathways to determine how much eugenol can be obtained from ferulic acid. *E. coli* NEB5α was transformed with the plasmid that contains the eugenol biosynthesis pathway (SBC009876) in combination with one of three selected plasmids that contain the coniferyl alcohol biosynthesis pathway (Fig. 3A). We selected the two top-performing pathway variants, SBC015866 and SBC015869, as well as SBC015863 for comparison. Strains were grown in shake flasks and eugenol production was monitored over the course of 72 h. A similar trend can be observed for the production of eugenol as was observed for coniferyl alcohol (Fig. 3B). The two top-performing coniferyl alcohol biosynthesis pathway candidates also yielded the most eugenol. The highest eugenol titre of 1.61 mM (264 mg/L) was obtained with the strain carrying SBC015866. Similar to the results of the combinatorial plasmid library screen, ferulic acid was fully consumed in the strains carrying SBC009876 in conjunction with either SBC015866 or SBC015869, whereas 25% of substrate remained unconverted in *E. coli* NEB5α SBC009876 SBC015863. Neither coniferyl aldehyde nor coniferyl alcohol were detected in any of the strains.

The eugenol production level of 264 mg/L represents a noteworthy enhancement compared to the previous production level of 102 mg/L achieved through the utilisation of SBC009876 in conjunction with a CoA-dependent coniferyl alcohol biosynthesis pathway [10]. This demonstrates a significant improvement in the production efficiency of eugenol, highlighting the potential of the CAR-dependent pathway.

Moreover, to validate the production of eugenol at a larger scale and in a medium more suitable for industrial processes, we performed a fed-batch fermentation of the best performing strain, *E. coli* NEB5α SBC015866 SBC009876, in a glucose minimal medium supplemented with 0.4% glycerol and 3 mM ferulic acid, utilising a 0.25 L bioreactor. After 72 h from inoculation, we achieved a maximum eugenol titre of 0.51 mM (83 mg/L), representing a slight increase over the eugenol titre of 0.49 mM (80 mg/L) attained after 24 h. Notably, since IPTG was added to the culture at the 24-hour time point to induce pathway expression, this suggests that the eugenol biosynthesis pathway had already been adequately expressed even in the absence of inducer, similar to the coniferyl alcohol production observed during the combinatorial plasmid library screen (Fig. 2B). This finding aligns with the decline in growth rate after the initial batch phase, as indicated by the CO_2_ production rate (Additional file 1: Figure S3), suggesting an accumulation of growth-inhibiting metabolites. Although the maximum eugenol titre of 83 mg/L is noticeably lower than the titre achieved in shake flasks, optimising process parameters, including media components and glucose feeding, as well as the removal of the product during cultivation, has the potential to further increase eugenol production at a larger scale.

### Leveraging enzyme promiscuity for the biosynthesis of diverse (hydroxy)cinnamyl alcohols and allylphenols

The enzymes used in this study have demonstrated broad substrate acceptance within phenylpropanoid metabolism. In addition to SrCAR and MsCAD2, which can convert various (hydroxy)cinnamic acids and (hydroxy)cinnamyl aldehydes, respectively, the enzymes employed for the two-step conversion of coniferyl alcohol into eugenol also exhibit wide substrate tolerance. For example, PhCFAT acetylates a diverse range of (hydroxy)cinnamyl alcohols, including cinnamyl alcohol, *p*-coumaryl alcohol, or sinapyl alcohol [16]. Similarly, ObEGS has been used for the in vivo reduction of *p*-coumaryl acetate [10] and in vitro conversion of sinapyl acetate into methoxyeugenol [18]. Given the promiscuity of these enzymes, we sought to harness the CAR-dependent pathway for the bioconversion of a range of (hydroxy)cinnamic acids into their corresponding (hydroxy)cinnamyl alcohols and subsequently allylphenols and allylbenzene. Alongside eugenol biosynthesis, we investigated the bioconversion of *p*-coumaric acid, caffeic acid, cinnamic acid, and sinapic acid into chavicol, hydroxychavicol, allylbenzene, and methoxyeugenol, respectively.

For each substrate, we initially performed a combinatorial plasmid library screen to determine the optimal pathway variant for the conversion of the (hydroxy)cinnamic acid into the respective (hydroxy)cinnamyl alcohol. Subsequently, two or three of these candidate pathways were combined with the pathway that converts (hydroxy)cinnamyl alcohols into allylphenols (SBC009876), as it was done for eugenol production. The *E. coli* NEB5α strain was employed for the bioconversion of *p*-coumaric acid, caffeic acid, and sinapic acid, while the *E. coli* NEB5α Δ*hcaE* strain, unable to catabolise cinnamic acid [26], was used for its bioconversion. *E. coli* NEB5α or *E. coli* NEB5α Δ*hcaE* carrying the nine pathway variants were supplemented with 3 mM (hydroxy)cinnamic acids and the biosynthesis of (hydroxy)cinnamyl alcohols was monitored for up to 48 h.

All four substrates were successfully converted into their respective alcohols, with maximum product titres of 2.71 mM *p*-coumaryl alcohol, 0.87 mM caffeyl alcohol, 0.7 mM cinnamyl alcohol, and 2.33 mM sinapyl alcohol (Table 2). In the case of *p*-coumaryl alcohol and cinnamyl alcohol, no residual substrate was detected in any of the strains after 16 and 24 h, respectively (Additional file 1: Figures S4 and S5). However, for caffeyl alcohol and sinapyl alcohol, significant levels of substrate remained unconverted for some pathway variants, while others showed more efficient conversion (Additional file 1: Figures S6 and S7). Among the pathway candidates, SBC015866 exhibited the most efficient substrate consumption, leaving only residual levels of caffeic acid and sinapic acid in the culture medium. The differences in conversion efficiency between pathway variants are also reflected in the accumulation of hydroxycinnamyl aldehyde intermediates. For *p*-coumaryl alcohol, certain pathway variants displayed significant levels of *p*-coumaraldehyde, reaching up to 0.98 mM, while pathway candidate SBC015866 maintained lower levels of this intermediate (Additional file 1: Figure S4).

Similar to the observations for coniferyl alcohol, the titres of *p*-coumaryl alcohol and sinapyl alcohol decreased over time (Additional file 1: Figures S4 and S7). A feeding experiment was conducted with all four (hydroxy)cinnamyl alcohols, revealing a decrease in concentration over time when fed to *E. coli* NEB5α grown in the culture medium (Additional file 1: Figure S8). Notably, even in the absence of cells, the titres of caffeyl alcohol and sinapyl alcohol decreased in the culture medium, indicating some instability of these compounds in the medium alone (Additional file 1: Figure S8). Interestingly, a colour change of the cells in the medium was observed for coniferyl alcohol, *p*-coumaryl alcohol, caffeyl alcohol, and sinapyl alcohol, ranging from yellow (*p*-coumaryl alcohol) to red (sinapyl alcohol) and brown (caffeyl alcohol) (Additional file 1: Figure S9). A similar colour change has been reported for coniferyl alcohol and sinapyl aldehyde as a result of peroxidase-mediated oxidative di- and polymerisation [27], suggesting an involvement of endogenous alcohol dehydrogenases and peroxidases in the metabolism of (hydroxy)cinnamyl alcohols in *E. coli* NEB5α. Nonetheless, despite their metabolism, the combinatorial plasmid library screen successfully identified the top-performing pathway candidate for each (hydroxy)cinnamyl alcohol (Table 2).

As demonstrated in the biosynthesis of eugenol from ferulic acid, the extension of the (hydroxy)cinnamyl alcohol biosynthesis pathway by PhCFAT and ObEGS (encoded by plasmid SBC009876) enabled the production of chavicol, hydroxychavicol, and methoxyeugenol from their respective hydroxycinnamic acid precursors (Table 2). The chavicol titre of 223 mg/L considerably surpasses the previously reported titre of 28 mg/L, which was achieved using a CoA-dependent pathway for the conversion of *p*-coumaric acid into *p*-coumaryl alcohol [10]. Moreover, the chavicol titre obtained here, through a four-step metabolic pathway starting with *p*-coumaric acid, is also significantly higher than the titre of 168 mg/L achieved previously by feeding 3 mM *p*-coumaryl alcohol to a strain solely expressing PhCFAT and ObEGS, which catalyse the last two steps [10]. This finding can be attributed to the rapid processing of intracellularly produced *p*-coumaryl alcohol, rather than the uptake and conversion of extracellularly supplemented *p*-coumaryl alcohol. To our knowledge, this is the first time that methoxyeugenol has been produced from sinapic acid in a microbial host, with a titre of 148 mg/L. Except for caffeic acid, which was fully consumed within 18 h (Additional file 1: Figure S10), less than 10% of *p*-coumaric acid and sinapic acid remained unconverted by the best-performing strain (Additional file 1: Figures S11 and S12). Additionally, hydroxycinnamyl alcohol intermediates were not detected for any allylphenol in the strains carrying SBC015866 or SBC015869 (Additional file 1: Figures S10, S11, and S12), indicating efficient in vivo processing into their respective hydroxycinnamyl acetates by PhCFAT. Moreover, once the maximum production was achieved, the allylphenol titres remained constant, highlighting the importance of rapid processing of the hydroxycinnamyl alcohol intermediate to avoid a decrease in product yield.

In contrast, allylbenzene could not be detected in *E. coli* NEB5α Δ*hcaE* carrying SBC009876 in combination with any of the three cinnamyl alcohol producing pathways (SBC015866, SBC015869, or SBC015871) and when supplemented with cinnamic acid. However, in all three strains, we observed the considerable accumulation of a compound that matches cinnamyl acetate based on its GC-MS/MS fragmentation pattern (Additional file 1: Figure S13). This suggests that although cinnamic acid is converted into cinnamyl acetate in vivo, ObEGS is unable to reduce it further to allylbenzene under the conditions tested in this study.

**Table 2:** (Hydroxy)cinnamyl alcohol and allylphenol titres achieved in this study. The pathway with which the titre was obtained and the sample time point are indicated. (−/+) indicates whether the culture was supplemented with IPTG. Titres represent the average of biological triplicates

Target compound	Highest titre obtained		Best-performing pathway (−/+ IPTG)	Time point (h)
	(mM)	(mg/L)		
*p*-Coumaryl alcohol	2.71 ± 0.08	407 ± 12	SBC015866 (+)	16
Caffeyl alcohol	0.87 ± 0.06	145 ± 9	SBC015867 (−)	24
Cinnamyl alcohol	0.70 ± 0.05	94 ± 7	SBC015871 (+)	24
Sinapyl alcohol	2.33 ± 0.08	490 ± 16	SBC015869 (−)	18
Chavicol	1.66 ± 0.06	223 ± 8	SBC015869 (+)	18
Hydroxychavicol	1.25 ± 0.15	188 ± 23	SBC015866 (+)	48
Methoxyeugenol	0.76 ± 0.01	148 ± 2	SBC015866 (+)	72

## Conclusion

In this study, we developed an optimised pathway for the in vivo bioconversion of various (hydroxy)cinnamic acids into their respective (hydroxy)cinnamyl alcohols. By extending this pathway, we successfully produced the allylphenols eugenol, chavicol, hydroxychavicol, and methoxyeugenol. Our production of eugenol and chavicol exceeds previously reported titres achieved using a CoA-dependent pathway for (hydroxy)cinnamic acid activation, while the biosynthesis of methoxyeugenol from sinapic acid represents a novel accomplishment. Importantly, we observed that *E. coli* NEB5α metabolised all (hydroxy)cinnamyl alcohols to a significant extent, highlighting the need for an optimised chassis strain and an efficient allylphenol biosynthesis pathway to minimise the accumulation of potential by-products. We believe that the CAR-dependent pathway holds broad potential for the production of (hydroxy)cinnamyl alcohols and other related allylphenols.

## Materials and methods

### Pathway and enzyme selection

RetroPath2.0 [28] was used to create potential biosynthetic pathways for the production of eugenol from ferulic acid (Additional file 1: Figure S14). To validate the pathway results, the reactions were cross-referenced with existing literature. Among the generated pathways, the one that sequentially converts ferulic acid into eugenol via coniferyl aldehyde, coniferyl alcohol, and coniferyl acetate was ultimately chosen based on its length, simplicity, and novelty. After selecting the pathway, Selenzyme [29] was employed to generate a list of enzyme candidates for each reaction in the proposed pathway. The final list of candidates was determined based on evidence from the literature, demonstrating successful expression and activity of the enzymes in *E. coli*. In cases where no ideal enzyme candidates were listed, alternative options were suggested after conducting a manual literature search.

### Base strains and media

New England Biolabs (NEB) 5α competent *E. coli* was used for cloning, plasmid propagation, and the biosynthesis of coniferyl alcohol, eugenol, *p*-coumaryl alcohol, chavicol, caffeyl alcohol, hydroxychavicol, sinapyl alcohol, and methoxyeugenol. Strain *E. coli* NEB5α Δ*hcaE* was employed to produce cinnamyl alcohol and allylbenzene. All strains used and generated in this study are listed in Additional file 1: Table S1. Bacterial cells were routinely grown in Luria-Bertani (LB) medium [30]. The in vivo bioconversion of (hydroxy)cinnamic acids into their corresponding (hydroxy)cinnamyl alcohols, allylphenols, and allylbenzene, as well as the evaluation of the chemical stability of (hydroxy)cinnamyl alcohols in the presence or absence of cells, were performed in phosphate-buffered Terrific Broth (TBP, Formedium) supplemented with 0.4% (w/v) glycerol. When required, antibiotics were added to the media at the following concentrations: 100 µg/mL carbenicillin, 34 µg/mL chloramphenicol, and 50 µg/mL kanamycin.

### Cloning and transformation

Plasmid DNA was extracted using the QIAprep Spin Miniprep Kit (Qiagen). DNA for cloning was amplified by PCR in 50 µL reactions using the Q5 High-Fidelity 2X Master Mix from NEB. Gel-purified linearised DNA was extracted using the Zymoclean Gel DNA Recovery Kit (Zymo Research). NEBuilder HiFi DNA Assembly Master Mix and restriction enzymes were purchased from NEB. Screening of bacterial colonies to confirm the chromosomal deletion of the *hcaE* gene was performed using the One*Taq* PCR Master Mix (2X, NEB) in 25 µL reactions. All PCR-, HiFi-, and digestion reactions were set up following the manufacturer’s instructions. Chemically competent *E. coli* were prepared and transformed by heat shock [30].

### Plasmid construction

Oligonucleotide primers were synthesised by Integrated DNA Technologies (IDT) and are listed in Additional file 1: Table S2. Gene parts were designed using PartsGenie, with RBS translation initiation rates set to 20,000 [31], and were custom-synthesised and cloned into pBbE2c-based expression plasmids [24] by TWIST Bioscience. Plasmids were constructed by HiFi DNA Assembly and a detailed summary of the constituent parts for each plasmid is provided in Additional file 1: Table S3. The correct assembly of plasmids was validated by Sanger sequencing (Eurofins Genomics). All plasmids used and generated in this study are listed in Additional file 1: Table S4. The nucleotide sequences of the plasmids SBC015863–SBC015871 have been deposited in the public version of the ACS registry (https://acs-registry.jbei.org) under the accession numbers ACS_000886–ACS_000894, respectively.

### Strain construction

The *E. coli* NEB5α *hcaE* deletion strain was constructed using CRISPR-based gene editing as reported previously [32]. For the construction of the target-specific vector, named pTF-hcaE, the *hcaE* gene was first screened for Cas12a protospacer adjacent motifs (PAMs) with the sequence TTTV. The CRISPR AsCpf1 insertion and deletion score web tool [33] was then used to evaluate the 27-bp long candidates of PAM and protospacer sequences, and the candidate with the highest score was selected for the construction of pTF-hcaE. The donor DNA, which contains 50 bp homologous arms, was designed to delete all but the start codon and the final seven codons at the 3′ end of the *hcaE* gene [32]. The deletion of *hcaE* was confirmed by colony PCR, using primers EHseq049 and EHseq050 that flank both sides of the recombination site.

### In vitro enzyme activity screening

The enzyme activity screening assays were performed following a previous protocol [10] with some modifications. The CAR and CAD enzymes were expressed directly from their respective pBbE2c-based expression plasmids, constructed by TWIST Bioscience, following the reported protocol [10]. Sfp was expressed from the pCDF1b_Sfp plasmid [14]. Crude lysates containing specific enzymes were directly used in the assays as follows: 100 µg of total protein crude lysate containing CAR and Sfp for the CAR assays, 25 µg of total protein crude lysate containing CAD/AKR for the CAD/AKR assays, and 250 µg of total protein crude lysate containing CAR and Sfp, along with 100 µg of total protein crude lysate containing CAD/AKR for the cascade assays. For the CAR assay, the reaction buffer consisted of 100 mM Tris-HCl (pH 7.5), 10 mM ATP, 10 mM MgCl_2_, 3 mM NADPH, and 3 mM ferulic acid in a 1-mL reaction volume. In the CAD/AKR assay, the reaction buffer contained 50 mM phosphate-buffered saline (pH 7.0), 0.2 mM NADPH, and 5 mM coniferyl aldehyde in a 1-mL reaction volume. The cascade assay buffer was the same as the CAR/AKR assay buffer, but with a higher NADPH concentration of 6 mM. All assays were incubated in 2-mL tubes at 30℃ with shaking at 250 rpm for 3 h before sampling and measuring the product titres using LC-MS/MS.

### Testing (hydroxy)cinnamyl alcohol stability

To assess the stability of different (hydroxy)cinnamyl alcohols in culture medium without cells, each alcohol was individually added to 1 mL of TBP medium in a 96-deepwell plate (DWP) to achieve a final concentration of 2 mM. The plate was then sealed with a breathable seal and incubated at 30℃ and 80% humidity with orbital shaking at 850 rpm. Samples were collected immediately after addition, as well as after 6 and 24 h.

To determine the stability of different (hydroxy)cinnamyl alcohols in culture medium containing cells, single colonies of *E. coli* NEB5α were used to inoculate 1 mL of TBP medium supplemented with glycerol (0.4% w/v) in a 96-DWP. The plate was also sealed with a breathable seal and incubated at 30℃ and 80% humidity with orbital shaking at 850 rpm for 16–18 h to grow the seed cultures. The main cultures were set up by diluting the seed cultures to an OD_600nm_ of 0.2 in 1 mL of fresh TBP medium supplemented with glycerol (0.4% w/v) and the respective (hydroxy)cinnamyl alcohol to a final concentration of 2 mM. Samples were collected immediately, as well as after 6 and 24 h. For both experiments, the (hydroxy)cinnamyl alcohol titres were quantified using either HPLC- or UPLC-DAD, depending on the specific target being analysed.

### Fed-batch fermentation

The fed-batch fermentation of *E. coli* NEB5α SBC015866 SBC009876 was performed in a 0.25 L bioreactor (DASbox® Mini Bioreactor System, Eppendorf). The fermentation medium consisted of 17.5 g/kg glucose, 6 g/kg KH_2_PO_4_, 5 g/kg (NH_4_)_2_SO_4_, 2 g/kg NaCl, 3 g/kg sodium citrate, 6 g/kg MgSO_4_ × 7H_2_O, 0.03 g/kg CaCl_2_, 0.15 g/kg thiamine HCl, 0.0225 g/kg FeSO_4_ × 7H_2_O, 0.5 g/kg yeast extract, and 3 g/kg trace element solution [34]. Additionally, the medium was supplemented with 0.4% glycerol, 3 mM ferulic acid, 50 mg/L kanamycin, and 34 mg/L chloramphenicol. The pH was maintained at 7.0 using 10% NH_4_OH, and the temperature was maintained at 37℃. The dissolved oxygen level was maintained at 40% through adjustment of the stirring rate, while a gas flow rate of 1 vvm was maintained. The bioreactor was inoculated with 2% biomass derived from a seed culture grown in shake flasks, and the initial glucose was consumed in a batch fermentation. Once the CO_2_ signal had dropped and the pH had increased to 7.15, a feed consisting of 55% (w/w) glucose was initiated. The feed rate was increased at an exponential rate of 0.1 h^− 1^ until an oxygen transfer rate of 180 mmol/kg/h was reached. Subsequently, a constant feed rate was maintained. The culture was induced by addition of IPTG to a final concentration of 0.1 mM, 24 h after inoculation.

### Biosynthesis of (hydroxy)cinnamyl alcohols, allylphenols, and allylbenzene

For the biosynthesis of (hydroxy)cinnamyl alcohols, allylphenols, and allylbenzene, single colonies of freshly transformed cells were used to inoculate 1 mL of TBP medium supplemented with glycerol (0.4% w/v) and the appropriate antibiotics in 96-DWP, which were sealed with breathable seals. The seed cultures were incubated at 30℃ and 80% humidity with orbital shaking at 850 rpm for 16–18 h.

To produce (hydroxy)cinnamyl alcohols or allylbenzene, main cultures were set up by diluting the seed cultures to an OD_600nm_ of 0.02 in 1 mL or 1.2 mL, respectively, of fresh TBP medium supplemented with glycerol (0.4% w/v) and the relevant antibiotics in 96-DWPs. These main cultures were returned to the shaker-incubator. For the biosynthesis of (hydroxy)cinnamyl alcohols, each seed culture was used to set up two main cultures: one remained uninduced, while the other was ultimately supplemented with isopropyl β-d-1-thiogalactopyranoside (IPTG).

To produce eugenol, chavicol, hydroxychavicol and methoxyeugenol, main cultures were prepared by diluting the seed cultures to an OD_600nm_ of 0.02 in 50 mL of fresh TBP medium supplemented with glycerol (0.4% w/v) and the relevant antibiotics in non-baffled 250-mL shake flasks. These cultures were incubated at 30℃ and 180 rpm.

To produce (hydroxy)cinnamyl alcohols, allylphenols, and allylbenzene, when the cultures reached an OD_600nm_ of 1.0–1.5, (hydroxy)cinnamic acids and optionally IPTG were added to final concentrations of 3 mM and 100 µM, respectively. Except for allylbenzene, the cultures were then returned to their respective shaker-incubator and grown for up to 72 h.

For the biosynthesis of allylbenzene, after the addition of cinnamic acid and IPTG, 1 mL of culture was transferred to a 20-mL headspace vial. The culture was overlaid with 0.5 mL of 2,2,4-trimethylpentane (TMP) supplemented with *sec*-butylbenzene (Sigma) to a final concentration of 0.005% (v/v). The vials were sealed with gas-tight screw caps and the cultures were incubated at 30℃ and 180 rpm for 24 h.

### Quantification of target compounds

For the quantification of (hydroxy)cinnamyl alcohols, allylphenols, and allylbenzene, as well as their precursor and intermediate compounds, different methods and instruments were employed. For compound quantification using HPLC-DAD, UPLC-DAD, and LC-MS/MS, culture samples were quenched by adding an equal volume of 100% methanol, followed by vortexing and centrifugation at 16,000 *g* for 10 min. For HPLC-DAD and UPLC-DAD analysis, cell-free supernatants were diluted 10-fold, while for LC-MS/MS analysis, cell free-supernatants were diluted 1000-fold using methanol/water (10:90 v/v). To quantify allylbenzene using GC-MS/MS, the organic layer was transferred to a 2-mL Eppendorf tube and centrifuged at 16,000 *g* for 10 min. The organic layer was then diluted 5-fold using TMP, vortexed with anhydrous Na_2_SO_4_ to remove residual water and transferred to a GC vial for analysis. The TMP used for sample dilution was supplemented with 0.005% *sec*-butylbenzene, which served as an internal standard. Metabolite concentrations were determined using calibration curves generated from standards of known concentrations, prepared in the same manner as the samples. A summary of the quantification method and the instrument used for each target compound can be found in the Supplementary Methods in Additional file 1.

### Electronic supplementary material

Below is the link to the electronic supplementary material.

Supplementary Material 1

## Data Availability

The datasets used and analysed during the current study are available from the corresponding author on reasonable request.
