# Supplementary material for: Carboxylic acid reductase-dependent biosynthesis of eugenol and related allylphenols
Source: Microb Cell Fact. 2023 Nov 18;22:238. doi: 10.1186/s12934-023-02246-4 (PMC10656918; doi:10.1186/s12934-023-02246-4)
Supplement: Supplementary file 1 — Supplementary Material 1 [file 12934_2023_2246_MOESM1_ESM.pdf]

Supplementary Information

Carboxylic acid reductase-dependent biosynthesis of eugenol and related allylphenols

Erik K. R. Hanko[1†], Kris Niño G. Valdehuesa[1†], Koen J. A. Verhagen[2], Jakub Chromy[1], Ruth A. Stoney[1], Jeremy Chua[1], Cunyu Yan[1], Johannes A. Roubos[2], Joep Schmitz[2], Rainer Breitling[1*]

[1]Manchester Institute of Biotechnology, Faculty of Science and Engineering, University of Manchester, 131 Princess Street, Manchester M1 7DN, United Kingdom

[2]dsm-firmenich, Science & Research, P.O. Box 1, 2600 MA Delft, The Netherlands

[†]These authors contributed equally to this work and share first authorship

[*]Author to whom correspondence should be addressed; E-Mail: rainer.breitling@manchester.ac.uk

Supplementary Methods

Quantification of target compounds

HPLC-ELSD analysis

Analytes from the eugenol biosynthesis experiment, including *ferulic acid*, *coniferaldehyde*, *coniferyl alcohol*, and *eugenol*, were quantified using high-performance liquid chromatography (HPLC). HPLC analysis was performed using an Agilent 1260 Infinity II LC system equipped with an evaporative light scattering detector (ELSD) measuring absorbance in the range of 190–600 nm. For analyte separation, an Agilent InfinityLab Poroshell 120 EC-C18 column (100 mm x 4.6 mm, 4 µm) was used at 50°C. The separation was achieved using a flow rate of 0.8 mL/min and a binary mobile phase consisting of A (H_2O, 0.05% NH_4OH) and B (MeOH, 0.05% NH_4OH). The gradient elution program was: 0–5 min, 99–40% A; 5–15 min, 40–2% A; 15–16 min, 2–99% A; 16–17 min, hold at 99% A. The injection volume was 5 µL. Peak areas were integrated using Agilent OpenLab software.

UPLC-DAD analysis

Ultra-performance liquid chromatography (UPLC) analysis was performed using an Agilent 1290 Infinity II LC system equipped with a diode array detector (DAD) measuring absorbance in the range of 190–640 nm. The mobile phases, column type, column temperature, gradient program, and absorbance wavelengths used were optimised to enable the best possible separation of analytes and are specified below. All samples were kept at 10°C throughout the analysis and the injection volume was 5 µL. Peak areas were integrated using Agilent OpenLab software.

p-Coumaric acid, p-coumaraldehyde, p-coumaryl alcohol, chavicol

A Waters Acquity HSS T3 column (50 mm x 2.1 mm, 1.8 µm) was used at 40°C. The separation was achieved using a flow rate of 0.6 mL/min and a binary mobile phase consisting of A (H_2O, 0.05% NH_4OH) and B (MeOH, 0.05% NH_4OH). The gradient elution program was: 0–0.5 min, 99–35% A; 0.5–1.0 min, 35–2% A; 1.0–2.0 min, hold at 2% A; 2.0–2.1 min, 2–99% A; 2.1–3.0 min, hold at 99% A.

Caffeic acid, caffeyl alcohol, hydroxychavicol

A Waters Acquity BEH C18 column (50 mm x 2.1 mm, 1.7 µm) was used at 45°C. The separation was achieved using a flow rate of 0.6 mL/min and a binary mobile phase consisting of A (H_2O, 0.1% formic acid) and B (MeOH, 0.1% formic acid). The gradient elution program was: 0–1.5 min, 80–5% A; 1.5–2.5 min, 5–80% A; 2.5–3.0 min, hold at 80% A.

Cinnamic acid, cinnamaldehyde, cinnamyl alcohol

A Waters Acquity BEH C18 column (50 mm x 2.1 mm, 1.7 µm) was used at 45°C. The separation was achieved using a flow rate of 0.6 mL/min and a binary mobile phase consisting of A (H_2O, 0.1% formic acid) and B (MeOH, 0.1% formic acid). The gradient elution program was: 0–1.0 min, hold at 50% A; 1.0–2.0 min, 50–5% A; 2.0–2.5 min, 5–50% A; 2.5–2.9 min, hold at 50% A.

Sinapic acid, sinapaldehyde, sinapyl alcohol, methoxyeugenol

A Waters Acquity BEH C18 column (50 mm x 2.1 mm, 1.7 µm) was used at 45°C. The separation was achieved using a flow rate of 0.6 mL/min and a binary mobile phase consisting of A (H_2O, 0.1% formic acid) and B (MeOH, 0.1% formic acid). The gradient elution program was: 0–1.0 min, hold at 72% A; 1.0–3.5 min, 72–5% A; 3.5–4.5 min, 5–72% A; 4.5–4.9 min, hold at 72% A.

LC-MS/MS analysis

Analytes from the *in vitro* enzyme assays and the coniferyl alcohol library screen, including *ferulic acid*, *coniferaldehyde*, and *coniferyl alcohol*, were quantified using liquid chromatography tandem mass spectrometry (LC-MS/MS). LC-MS/MS analysis was performed as reported previously using a Waters ACQUITY UPLC H-Class System coupled to a Xevo TQ-S triple-quadrupole mass spectrometer

(Waters) equipped with an electrospray ionisation (ESI) source[1]. All analytes were monitored in ESI^{-} mode. The LC method and MS method parameters for each individual analyte, including precursor ion mass, product ion mass, cone voltage, and collision energy have been reported previously[1]. Peak areas were integrated using MassLynx v 4.1 (Waters).

GC-MS analysis

Allylbenzene was detected and analysed using gas chromatography mass spectrometry (GC-MS). GC-MS analysis was performed using an Agilent 7890B GC system coupled to a 7200 accurate mass Q-TOF mass spectrometer equipped with an electron impact ion source using 70 eV ionisation and a fixed emission of 35 µA. A VF-5ms column (30 m x 250 µm x 0.25 µm, Agilent Technologies) was used with an inlet temperature of 280°C and a split ratio of 100:1. Helium was used as the carrier gas with a flow rate of 1.5 mL/min and a pressure of 16.2 psi. The chromatography program was: 0–1.0 min, hold at 50°C; 1.0–5.29 min, 50–350°C; 5.29–6.29 min, hold at 350°C. The total runtime per analysis was 6.29 min. The sample (1 µL) was injected using a PAL RSI 85 autosampler. The mass spectrum was collected for the range of 50–500 m/z with an acquisition rate of 5 spectra/s and an acquisition time of 200 ms/spectrum.

Supplementary Tables

Table S1. Strains used and generated in this study.

Strain	Purpose	Reference or source
E. coli NEB5α	Biosynthesis of coniferyl alcohol, eugenol, *p*-coumaryl alcohol, chavicol, caffeyl alcohol, hydroxychavicol, sinapyl alcohol, and methoxyeugenol.	NEB
E. coli NEB5α Δ*hcaE*	Biosynthesis of cinnamyl alcohol and allylbenzene.	This study

Table S2. Oligonucleotide primers used in this study.

Primer name	Sequence (5′ to 3′)
001BsSfp-pBb-F_01	GAGCTTTTATAAGGATCCAAACTCGAGTAAGGA
002pBb-SrCar-R_01	ATTACCACAATGACTTCTTAAAAGATCTTTTGAATTCTGAAATTG
003pBb-SrCAR-F_01	ATCTTTTAAGAAGTCATTGTGGTAATATCCGATCCG
004SrCAR-MsCAD2-R_01	AAAACACAGCCTTATAACAGGCCCAGTTGTTTG
005SrCAR-MsCAD2-F_01	CCTGTTATAAGGCTGTGTTTTGAAATTAACCAC
006MsCAD2-BsSFp-R_01	CTCCTTATTAAAGTTCACTCTTCAAATTTGCTGC
007MsCAD2-BsSfp-F_01	ATTTGAAGAGTGAACTTTAATAAGGAGATATACCATGGG
008BsSfp-pBb-R_01	GAGTTTGGATCCTTATAAAAGCTCTTCGTACGAGACC
009pBb-MsCAD2-R_01	AAAACACAGCCCTTCTTAAAAGATCTTTTGAATTCTGA
010pBb-MsCAD2-F_01	TTTTAAGAAGGGCTGTGTTTTGAAATTAACC
011MsCAD2-SBC0126-R_01	CTGCCAGATATCACTCTTCAAATTTGCTGC
012MsCAD2-SBC0126-F_01	TGAAGAGTGATATCTGGCAGTCAGGGATT
013SBC0126-SrCAR-R_01	TACCACAATGAGTGAGTGCAGGTTATCTACG
014SBC0126-SrCAR-F_01	CTGCACTCACTCATTGTGGTAATATCCGATCCG
015SrCAR-SBC0127-R_01	TCCTACGCGATTATAACAGGCCCAGTTGT
016SrCAR-SBC0127-F_01	CCTGTTATAATCGCGTAGGATTGTGG
017SBC0127-BsSfp-R_01	CTTATTAAAGTTGCTGGATACGACGCT
018SBC0127-BsSfp-F_01	CGTATCCAGCAACTTTAATAAGGAGATATACCATGGG
019MsCAD2-pBb-F_01	TGAAGAGTGAGGATCCAAACTCGAGTAAG
020pBb-BsSfp-R_01	CTCCTTATTAAAGTCTTCTTAAAAGATCTTTTGAATTCTGAAATTG
021pBb-BsSfp-F_01	GATCTTTTAAGAAGACTTTAATAAGGAGATATACCATGGG
022BsSfp-SrCAR-R_01	TATTACCACAATGATTATAAAAGCTCTTCGTACGAGAC
023BsSfp-SrCAR-F_01	AGAGCTTTTATAATCATTGTGGTAATATCCGATCC
024MsCAD2-pBb-R_01	GTTTGGATCCTCACTCTTCAAATTTGCTGC
025SrCAR-pBb-F_01	CCTGTTATAAGGATCCAAACTCGAGTAAG

026BsSfp-MsCAD2-R_01	AAAACACAGCCTTATAAAAGCTCTTCGTACGAGACC
027BsSfp-MsCAD2-F_01	AGCTTTTATAAGGCTGTGTTTTGAAATTAACCAC
028MsCAD2-SrCAR-R_01	TACCACAATGATCACTCTTCAAATTTGCTGC
029MsCAD2-SrCAR-F_01	TGAAGAGTGATCATTGTGGTAATATCCG
030SrCAR-pBb-R_01	AGTTTGGATCCTTATAACAGGCCCAGTTGT
031BsSfp-SBC0127-R-01	ATCCTACGCGATTATAAAAGCTCTTCGTACGAGAC
032BsSfp-SBC0127-F-01	AGCTTTTATAATCGCGTAGGATTGTGG
033SBC0127-SrCAR-R-01	TACCACAATGATGCTGGATACGACGC
034SBC0127-SrCAR-F-01	GTATCCAGCATCATTGTGGTAATATCCGATCCG
035SrCAR-SBC0126-R-01	ACTGCCAGATATTATAACAGGCCCAGTTGTT
036SrCAR-SBC0126-F-01	GCCTGTTATAATATCTGGCAGTCAGGGATT
037SBC0126-BsSfp-R-01	CTTATTAAAGTGTGAGTGCAGGTTATCTACG
038SBC0126-BsSfp-F-01	CCTGCACTCACACTTTAATAAGGAGATATACCATGGG
039BsSfp-SBC0124-R-01	TGAGCCCAATCTTATAAAAGCTCTTCGTACGAGACC
040BsSfp-SBC0127-F-01	AGCTTTTATAAGATTGGGCTCAACTTCTACG
041SBC0124-MsCAD2-R-01	AAACACAGCCCCTGCGTTTACCGTGAG
042SBC0124-MsCAD2-F-01	TAAACGCAGGGGCTGTGTTTTGAAATTAACC
043MsCAD2-SBC0088-R-01	AAGGGAACTATTCACTCTTCAAATTTGCTGC
044MsCAD2-SBC0088-F-01	TTGAAGAGTGAATAGTTCCCTTCACGATAGC
045SBC0088-BsSfp-R-01	CTTATTAAAGTCGTCTTCGCTTCCCTAC
046SBC0088-BsSfp-F-01	AAGCGAAGACGACTTTAATAAGGAGATATACCATGGG
047SrCAR-SBC0124-R-01	TGAGCCCAATCTTATAACAGGCCCAGTTGTTTG
048SrCAR-SBC0124-F-01	CCTGTTATAAGATTGGGCTCAACTTCTACG
049SBC0124-BsSfp-R-01	CCTTATTAAAGTCCTGCGTTTACCGTGAG
050SBC0124-BsSfp-F-01	GTAAACGCAGGACTTTAATAAGGAGATATACCATGGG
EH210_f	AATTTCTACTCTTGTAGATCCAACTGATTGACACCCAAAATGTTTTTTTGAAGCTTGGGCCC
EH211_r	CCTGCTGCATATTTTTTAATCCTGAAAGATAAGATGTATTGACCTTCTATTTTTGTGCGTACCCTAGGTATAAACGCAG
EH212_f	AAAATATGCAGCAGGAGGTGATGAAATGAGTGCGCAAGTTTCACTAGAGTTACATCACTGAATTCTCTAGAGTCGACCTG
EH213_r	GGATCTACAAGAGTAGAAATTACTAGTATTATACCTAGGACTGAGCTAG
EHseq049	GCGACGAAATAGCGTAAATGC
EHseq050	CGTGTTTGCGCGTTAACTG

Table S3. Constituent parts used for plasmid assembly.

Plasmid	Part	Forward primer	Reverse primer	Template
pTF-hcaE	Backbone	EH212_f	EH213_r	pTF
	Guide RNA and donor DNA	EH210_f	EH211_r	pTF
SBC015863	pBbA1c	001BsSfp-pBb-F_01	009pBb-MsCAD2-R_01	pBbA1c-rfp
	MsCAD2	010pBb-MsCAD2-F_01	011MsCAD2-SBC0126-R_01	SBC013260
	SBC0126	012MsCAD2-SBC0126-F_01	013SBC0126-SrCAR-R_01	SBC009918
	SrCAR	014SBC0126-SrCAR-F_01	015SrCAR-SBC0127-R_01	SBC013258
	SBC0127	016SrCAR-SBC0127-F_01	017SBC0127-BsSfp-R_01	SBC004928
	BsSfp	018SBC0127-BsSfp-F_01	008BsSfp-pBb-R_01	pCDF1b_Sfp
SBC015864	pBbA5c	001BsSfp-pBb-F_01	002pBb-SrCar-R_01	pBbA5c-rfp
	SrCAR	003pBb-SrCAR-F_01	004SrCAR-MsCAD2-R_01	SBC013258
	MsCAD2	005SrCAR-MsCAD2-F_01	006MsCAD2-BsSFp-R_01	SBC013260
	BsSfp	007MsCAD2-BsSfp-F_01	008BsSfp-pBb-R_01	pCDF1b_Sfp
SBC015865	pBbE5c	019MsCAD2-pBb-F_01	020pBb-BsSfp-R_01	pBbE5c-rfp
	BsSfp	021pBb-BsSfp-F_01	022BsSfp-SrCAR-R_01	pCDF1b_Sfp
	SrCAR	023BsSfp-SrCAR-F_01	004SrCAR-MsCAD2-R_01	SBC013258
	MsCAD2	005SrCAR-MsCAD2-F_01	024MsCAD2-pBb-R_01	SBC013260
SBC015866	pBbE1c	025SrCAR-pBb-F_01	020pBb-BsSfp-R_01	pBbE1c-rfp
	BsSfp	021pBb-BsSfp-F_01	026BsSfp-MsCAD2-R_01	pCDF1b_Sfp
	MsCAD2	027BsSfp-MsCAD2-F_01	028MsCAD2-SrCAR-R_01	SBC013260
	SrCAR	029MsCAD2-SrCAR-F_01	030SrCAR-pBb-R_01	SBC013258
SBC015867	pBbE5c	025SrCAR-pBb-F_01	009pBb-MsCAD2-R_01	pBbE5c-rfp
	MsCAD2	010pBb-MsCAD2-F_01	006MsCAD2-BsSFp-R_01	SBC013260
	BsSfp	007MsCAD2-BsSfp-F_01	031BsSfp-SBC0127-R-01	pCDF1b_Sfp
	SBC0127	032BsSfp-SBC0127-F-01	033SBC0127-SrCAR-R-01	SBC004928
	SrCAR	034SBC0127-SrCAR-F-01	030SrCAR-pBb-R_01	SBC013258
SBC015868	pBbE5c	019MsCAD2-pBb-F_01	002pBb-SrCar-R_01	pBbE5c-rfp
	SrCAR	003pBb-SrCAR-F_01	035SrCAR-SBC0126-R-01	SBC013258
	SBC0126	036SrCAR-SBC0126-F-01	037SBC0126-BsSfp-R-01	SBC009918
	BsSfp	038SBC0126-BsSfp-F-01	039BsSfp-SBC0124-R-01	pCDF1b_Sfp
	SBC0124	040BsSfp-SBC0127-F-01	041SBC0124-MsCAD2-R-01	SBC009968
	MsCAD2	042SBC0124-MsCAD2-F-01	024MsCAD2-pBb-R_01	SBC013260
SBC015869	pBbE1c	025SrCAR-pBb-F_01	009pBb-MsCAD2-R_01	pBbE1c-rfp
	MsCAD2	010pBb-MsCAD2-F_01	043MsCAD2-SBC0088-R-01	SBC013260
	SBC0088	044MsCAD2-SBC0088-F-01	045SBC0088-BsSfp-R-01	SBC009968
	BsSfp	046SBC0088-BsSfp-F-01	022BsSfp-SrCAR-R_01	pCDF1b_Sfp
	SrCAR	023BsSfp-SrCAR-F_01	030SrCAR-pBb-R_01	SBC013258
SBC015870	pBbA1c	025SrCAR-pBb-F_01	009pBb-MsCAD2-R_01	pBbA1c-rfp
	MsCAD2	010pBb-MsCAD2-F_01	043MsCAD2-SBC0088-R-01	SBC013260
	SBC0088	044MsCAD2-SBC0088-F-01	045SBC0088-BsSfp-R-01	SBC009968
	BsSfp	046SBC0088-BsSfp-F-01	022BsSfp-SrCAR-R_01	pCDF1b_Sfp
	SrCAR	023BsSfp-SrCAR-F_01	030SrCAR-pBb-R_01	SBC013258
SBC015871	pBbA5c	001BsSfp-pBb-F_01	009pBb-MsCAD2-R_01	pBbA5c-rfp
	MsCAD2	010pBb-MsCAD2-F_01	028MsCAD2-SrCAR-R_01	SBC013260
	SrCAR	029MsCAD2-SrCAR-F_01	047SrCAR-SBC0124-R-01	SBC013258
	SBC0124	048SrCAR-SBC0124-F-01	049SBC0124-BsSfp-R-01	SBC009968
	BsSfp	050SBC0124-BsSfp-F-01	008BsSfp-pBb-R_01	pCDF1b_Sfp

Table S4. Plasmids used and generated in this study.

Plasmid	Characteristic	Reference or source
pBbA1c-rfp	Cm^R; p15A; P_{trc}-rfp	[2]
pBbA5c-rfp	Cm^R; p15A; P_{lacUV5}-rfp	[2]
pBbE1c-rfp	Cm^R; ColE1; P_{trc}-rfp	[2]
pBbE5c-rfp	Cm^R; ColE1; P_{lacUV5}-rfp	[2]
pSIMcpf1	Vector for CRISPR-Cas12a-mediated genome engineering	[3]
pTF	Vector for CRISPR-Cas12a-mediated genome editing	[3]
pCDF1b_Sfp	$Spec^R$; CloDF13; P-Sfp	[4]
SBC009904	Cm^R; ColE1; P_{trc}-ZmCCR2-TB1006-P_{trc}-PsCAD-TB1006-P_{lacUV5}-Gm4CL3	[1]
SBC009876	Kan^R; pBBR1; P_{trc}-PhCFAT-ObEGS	[1]
SBC009918	Cm^R; ColE1; P_{trc}-MtCCR1-TB1006-P_{lacUV5}-Gm4CL3-PsCAD	[1]
SBC009968	Cm^R; ColE1; P_{trc}-PsCAD-TB1006-P_{trc}-Gm4CL3-TB1006-PhCCR1	[1]
pTF-hcaE	Sm^R; ColE1; *hcaE* spacer and donor DNA	This study
SBC013256	Cm^R; ColE1; P_{tet}-NiCAR	This study
SBC013258	Cm^R; ColE1; P_{tet}-SrCAR	This study
SBC016024	Cm^R; ColE1; P_{tet}-PsCAD	This study
SBC013260	Cm^R; ColE1; P_{tet}-MsCAD2	This study
SBC013262	Cm^R; ColE1; P_{tet}-CgAKR1	This study
SBC015863	Cm^R; p15A; P_{trc}-MsCAD2, P_{lacUV5}-SrCAR, P_{lacUV5}-BsSfp	This study
SBC015864	Cm^R; p15A; P_{lacUV5}-SrCAR-MsCAD2-BsSfp	This study
SBC015865	Cm^R; ColE1; P_{lacUV5}-BsSfp-SrCAR-MsCAD2	This study
SBC015866	Cm^R; ColE1; P_{trc}-BsSfp-MsCAD2-SrCAR	This study
SBC015867	Cm^R; ColE1; P_{lacUV5}-MsCAD2-BsSfp, P_{lacUV5}-SrCAR	This study
SBC015868	Cm^R; ColE1; P_{lacUV5}-SrCAR, P_{lacUV5}-BsSfp, P_{trc}-MsCAD2	This study
SBC015869	Cm^R; ColE1; P_{trc}-MsCAD2, P_{trc}-BsSfp-SrCAR	This study
SBC015870	Cm^R; p15A; P_{trc}-MsCAD2, P_{trc}-BsSfp-SrCAR	This study
SBC015871	Cm^R; p15A; P_{lacUV5}-MsCAD2-SrCAR, P_{trc}-BsSfp	This study

Supplementary Figures

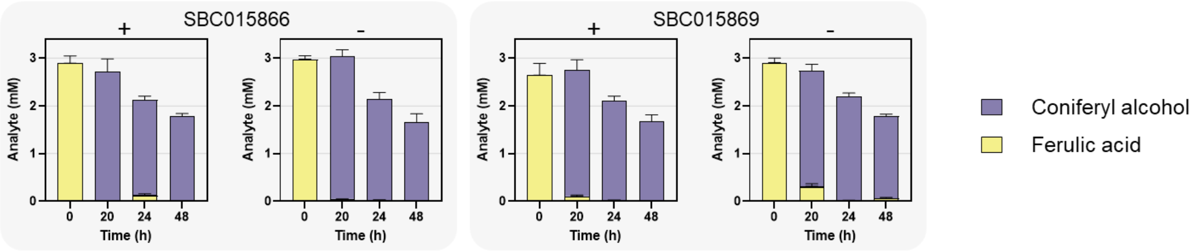

Figure S1. Production of coniferyl alcohol from ferulic acid. *E. coli* NEB5α carrying plasmid SBC015866 or SBC015869 was grown at 30°C in TBP medium supplemented with 0.4% glycerol and 3 mM ferulic acid. Cells were grown in the absence (−) or presence (+) of IPTG at a final concentration of 100 µM. Data represent mean and standard deviations of three biological replicates.

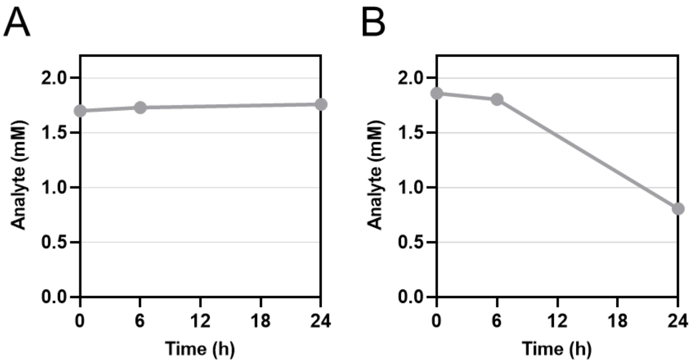

Figure S2. Stability of coniferyl alcohol in TBP medium **A** in the absence and **B** presence of *E. coli* NEB5α cells. The stability assay was performed in TBP medium supplemented with coniferyl alcohol at a final concentration of 2 mM. In case of **B**, the medium also contained 0.4% glycerol, and *E. coli* NEB5α cells were added at t=0 to achieve an OD_{600nm} of 0.2. The assays were carried out in 96-deepwell plates (DWP), sealed with breathable seals, at 30°C, 80% humidity, and orbital shaking at 850 rpm. For **A**, values represent single replicates. For **B**, values represent the mean of two replicates.

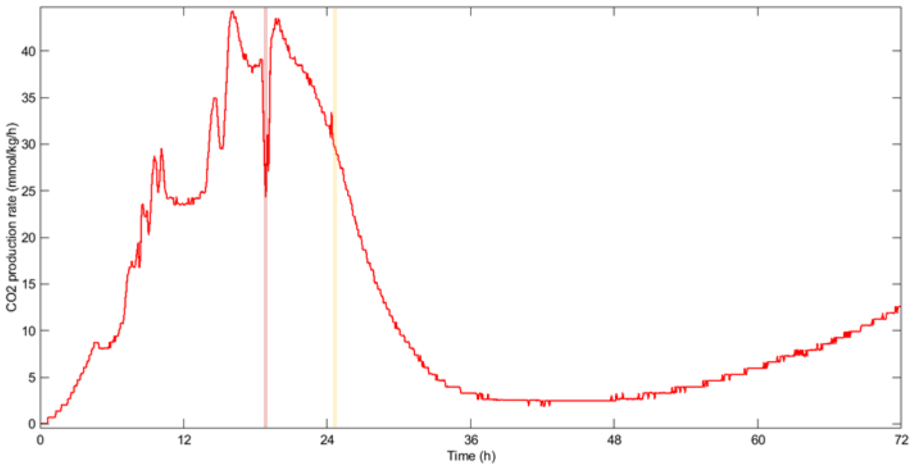

Figure S3. CO_2 production rate over the course of cultivation. The red vertical line at 19.1 h indicates the initiation of the glucose feed. The yellow vertical line indicates the induction with IPTG.

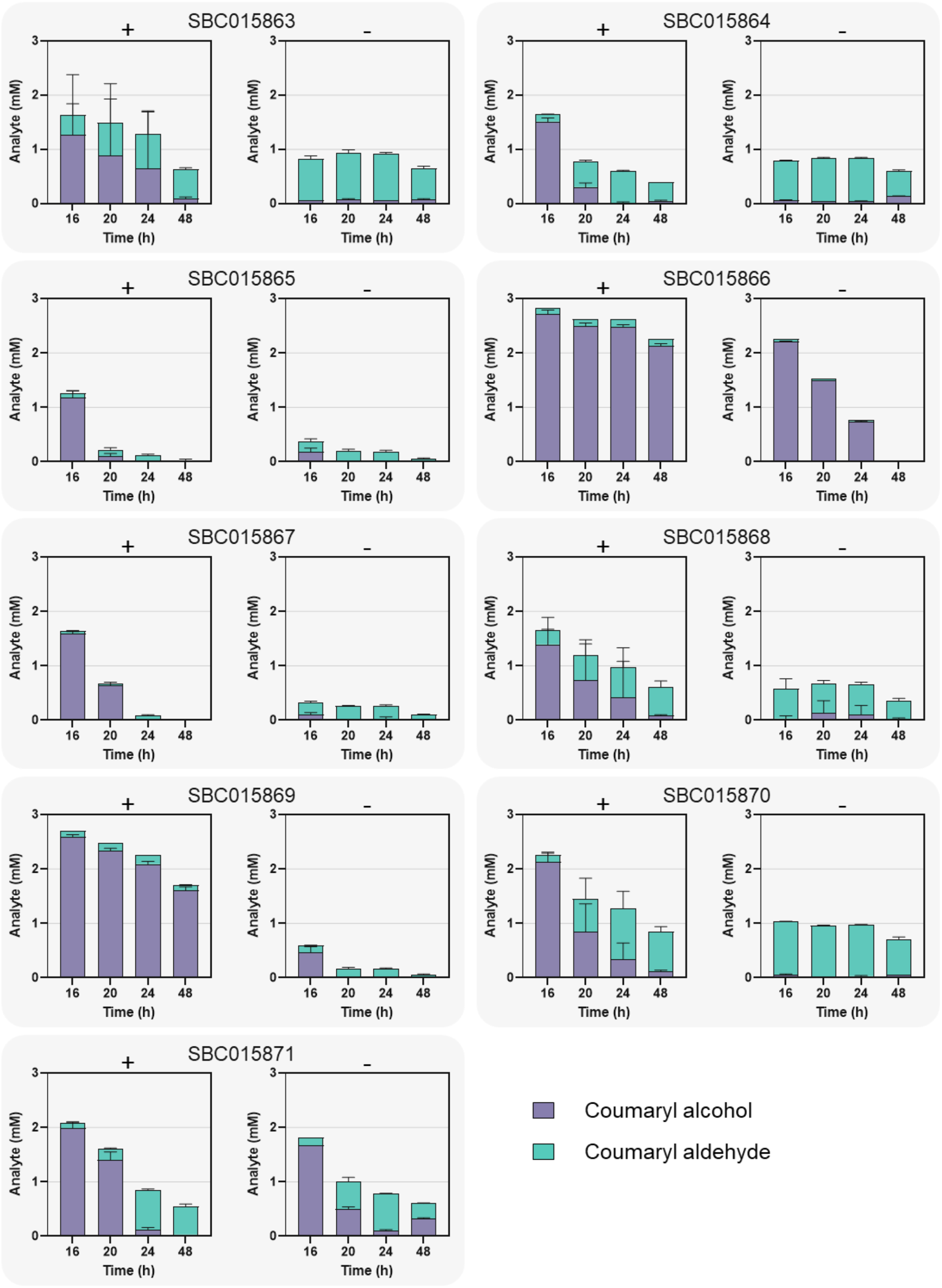

Figure S4. Production of *p*-coumaryl alcohol from *p*-coumaric acid. *E. coli* NEB5α carrying plasmids SBC015863–SBC015871 were grown at 30°C in TBP medium supplemented with 0.4% glycerol and 3 mM *p*-coumaric acid. Cells were grown in the absence (−) or presence (+) of IPTG at a final concentration of 100 µM. Error bars represent standard deviations of three biological replicates.

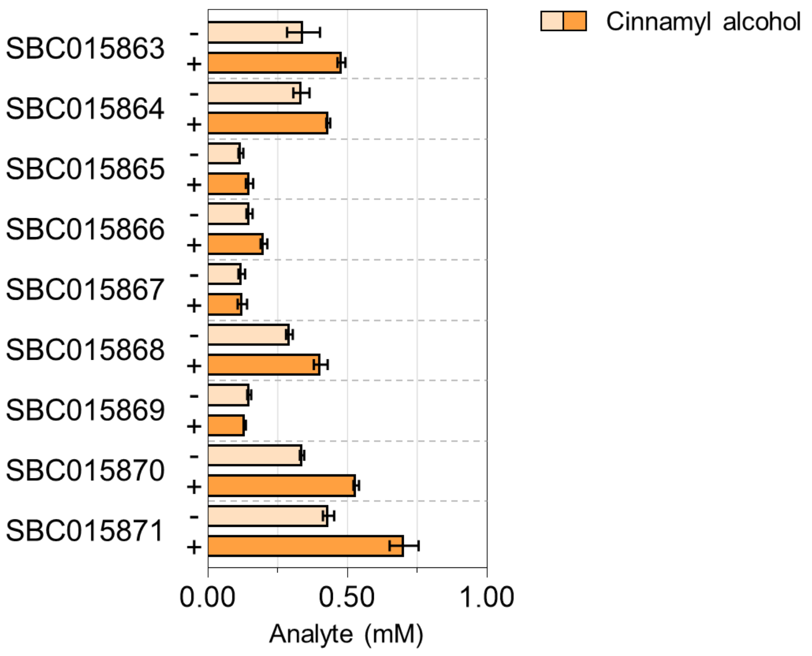

Figure S5. Production of cinnamyl alcohol from cinnamic acid. *E. coli* NEB5α carrying plasmids SBC015863–SBC015871 were grown at 30°C in TBP medium supplemented with 0.4% glycerol and 3 mM cinnamic acid. Cells were grown in the absence (−) or presence (+) of IPTG at a final concentration of 100 µM. Samples were taken after 24 h. Error bars represent standard deviations of three biological replicates.

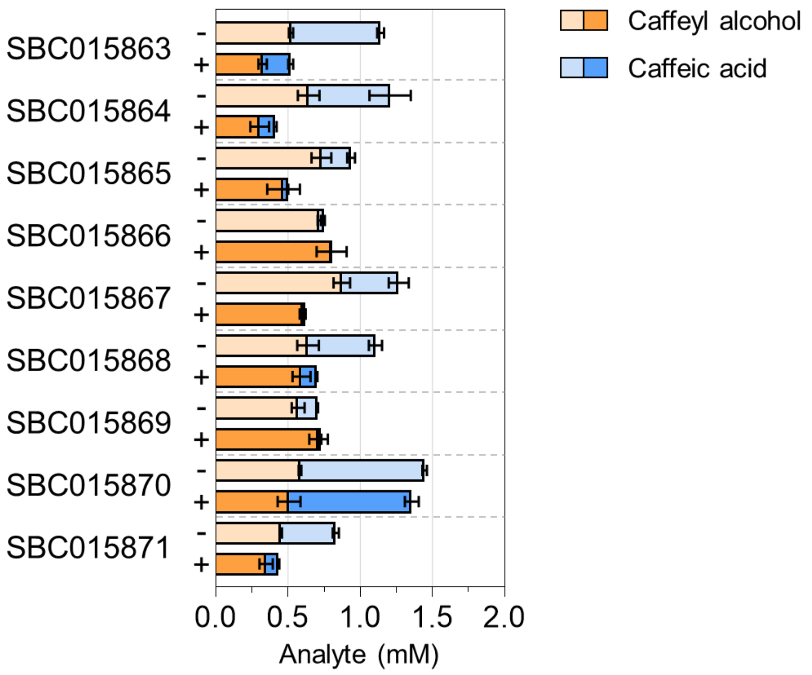

Figure S6. Production of caffeyl alcohol from caffeic acid. *E. coli* NEB5α carrying plasmids SBC015863–SBC015871 were grown at 30°C in TBP medium supplemented with 0.4% glycerol and 3 mM caffeic acid. Cells were grown in the absence (−) or presence (+) of IPTG at a final concentration of 100 µM. Samples were taken after 24 h. Error bars represent standard deviations of three biological replicates.

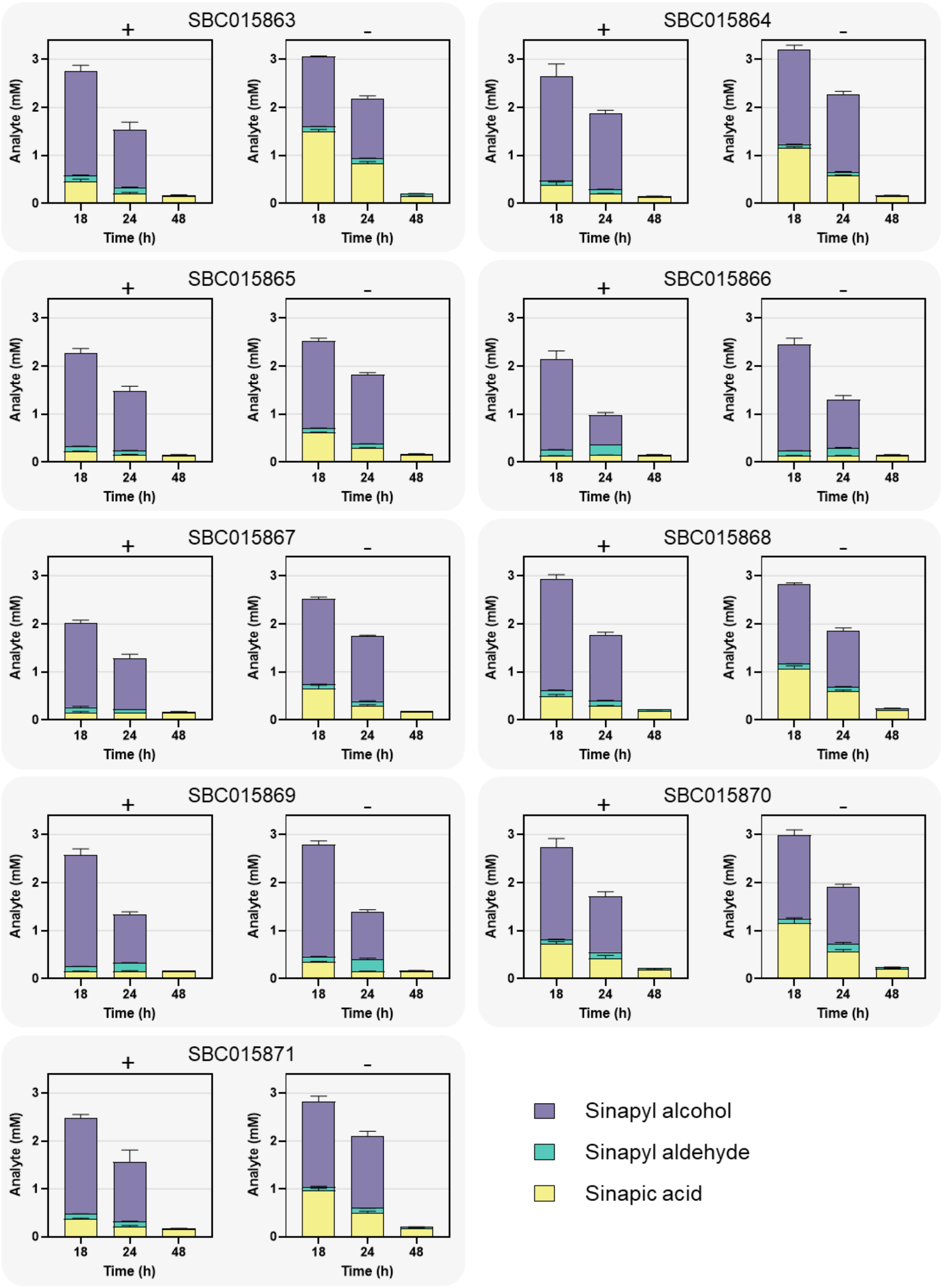

Figure S7. Production of sinapyl alcohol from sinapic acid. *E. coli* NEB5α carrying plasmids SBC015863–SBC015871 were grown at 30°C in TBP medium supplemented with 0.4% glycerol and 3 mM sinapic acid. Cells were grown in the absence (−) or presence (+) of IPTG at a final concentration of 100 µM. Error bars represent standard deviations of three biological replicates.

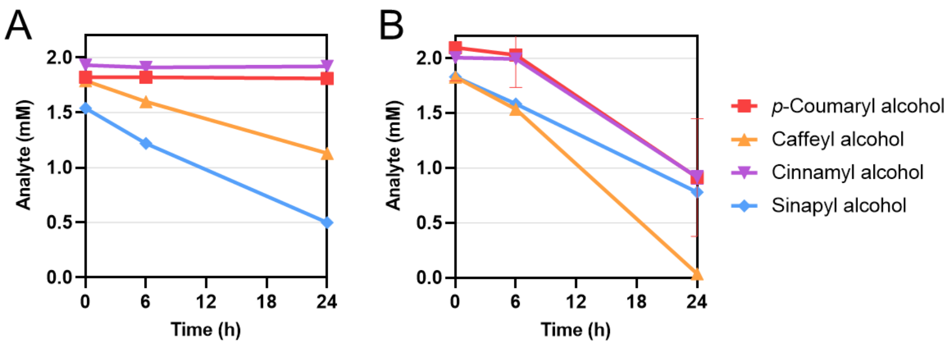

Figure S8. Stability of *p*-coumaryl alcohol, caffeyl alcohol, cinnamyl alcohol, and sinapyl alcohol **A** in TBP medium in the absence and **B** presence of *E. coli* NEB5α cells. The stability assay was performed in TBP medium supplemented with the respective alcohol at a final concentration of 2 mM. In case of **B**, the medium also contained 0.4% glycerol, and *E. coli* NEB5α cells were added at t=0 to achieve an OD_{600nm} of 0.2. The assays were carried out in 96-DWP, sealed with breathable seals, at 30°C, 80% humidity, and orbital shaking at 850 rpm. For **A**, values represent single replicates. For **B**, values represent the mean of two replicates.

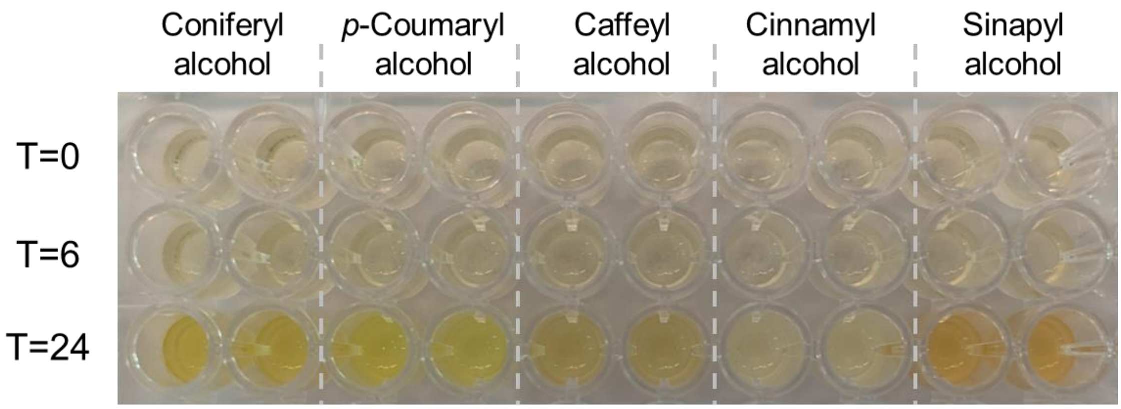

Figure S9. Stability of coniferyl alcohol, *p*-coumaryl alcohol, caffeyl alcohol, cinnamyl alcohol, and sinapyl alcohol in TBP medium supplemented with 0.4% glycerol and the respective alcohol at a final concentration of 2 mM. *E. coli* NEB5α cells were added at t=0 to reach an OD_{600nm} of 0.2. The cultures were grown in 96-DWP, sealed with breathable seals, at 30°C, 80% humidity, and orbital shaking at 850 rpm. Samples of 100 µL were collected immediately, as well as 6 and 24 h after addition of the cells. The samples were kept at −20°C until the last time point and then transferred to a clear 96-well microtitre plate for visualisation.

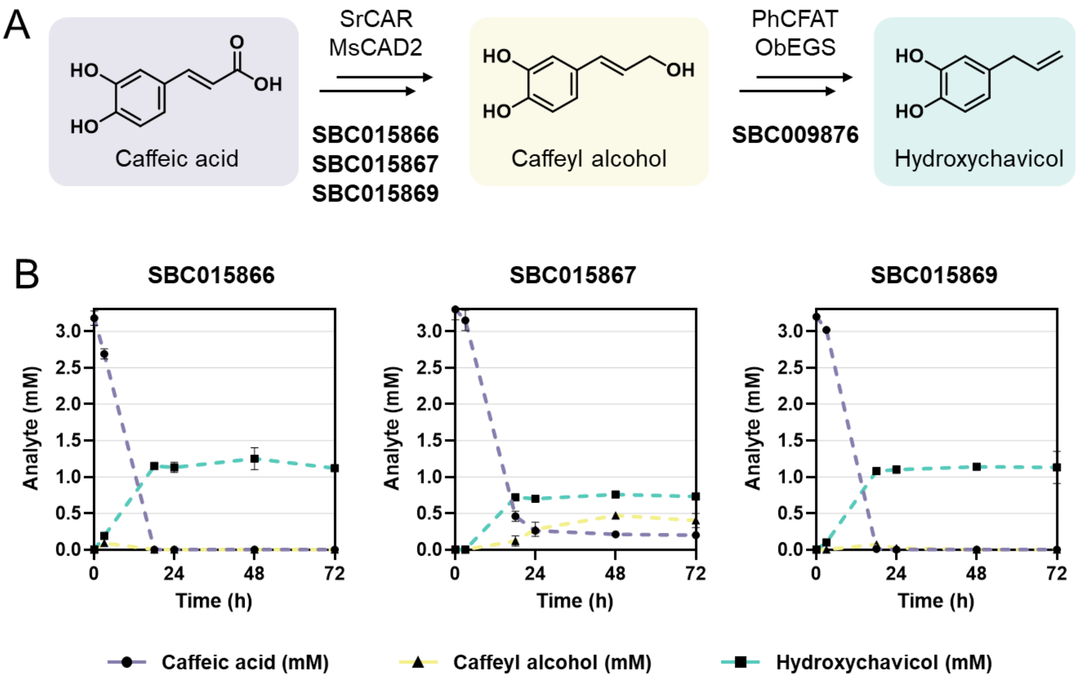

Figure S10. Bioconversion of caffeic acid into hydroxychavicol using the CAR-dependent pathway. **A** The hydroxychavicol biosynthesis pathway is split into two modules. The first module consists of SrCAR and MsCAD2 enzymes, catalysing the two-step reduction of caffeic acid, resulting in the formation of caffeyl alcohol. The second module comprises PhCFAT and ObEGS enzymes, catalysing the two-step conversion of caffeyl alcohol into hydroxychavicol. **B** Production of hydroxychavicol in *E. coli* NEB5α carrying SBC009876 in combination with one of the three indicated plasmids. At time point zero, cells were supplemented with 3 mM caffeic acid, and expression of pathway enzymes was induced by addition of IPTG. Error bars represent standard deviations of biological triplicates.

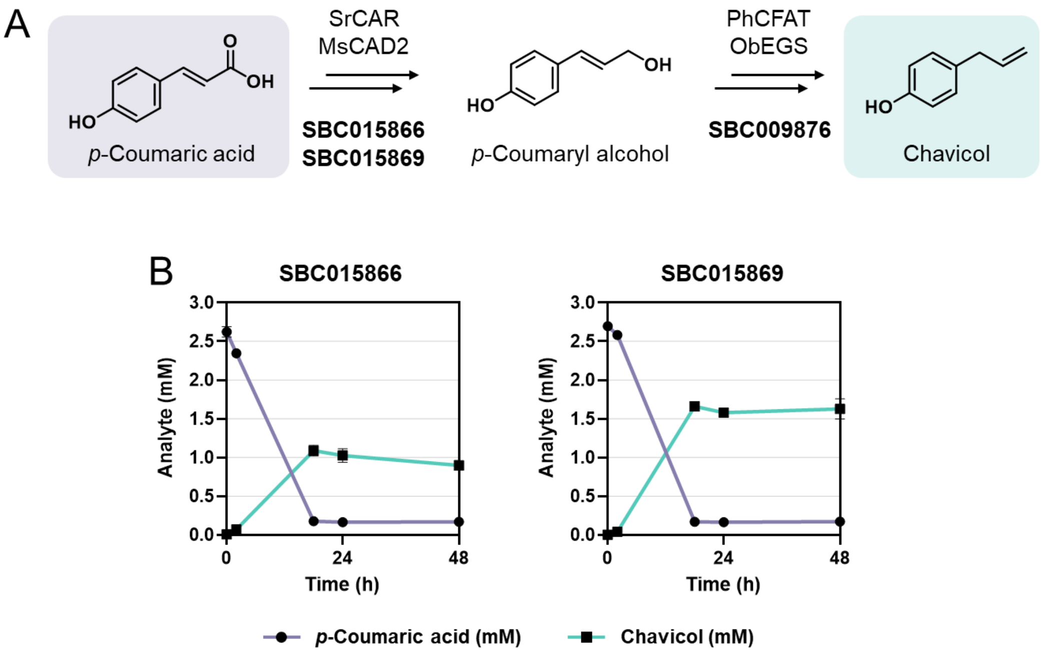

Figure S11. Bioconversion of *p*-coumaric acid into chavicol using the CAR-dependent pathway. **A** The chavicol biosynthesis pathway is split into two modules. The first module consists of SrCAR and MsCAD2 enzymes, catalysing the two-step reduction of *p*-coumaric acid, resulting in the formation of *p*-coumaryl alcohol. The second module comprises PhCFAT and ObEGS enzymes, catalysing the two-step conversion of *p*-coumaryl alcohol into chavicol. **B** Production of chavicol in *E. coli* NEB5α carrying SBC009876 in combination with one of the two indicated plasmids. At time point zero, cells were supplemented with 3 mM *p*-coumaric acid, and expression of pathway enzymes was induced by addition of IPTG. Error bars represent standard deviations of biological triplicates.

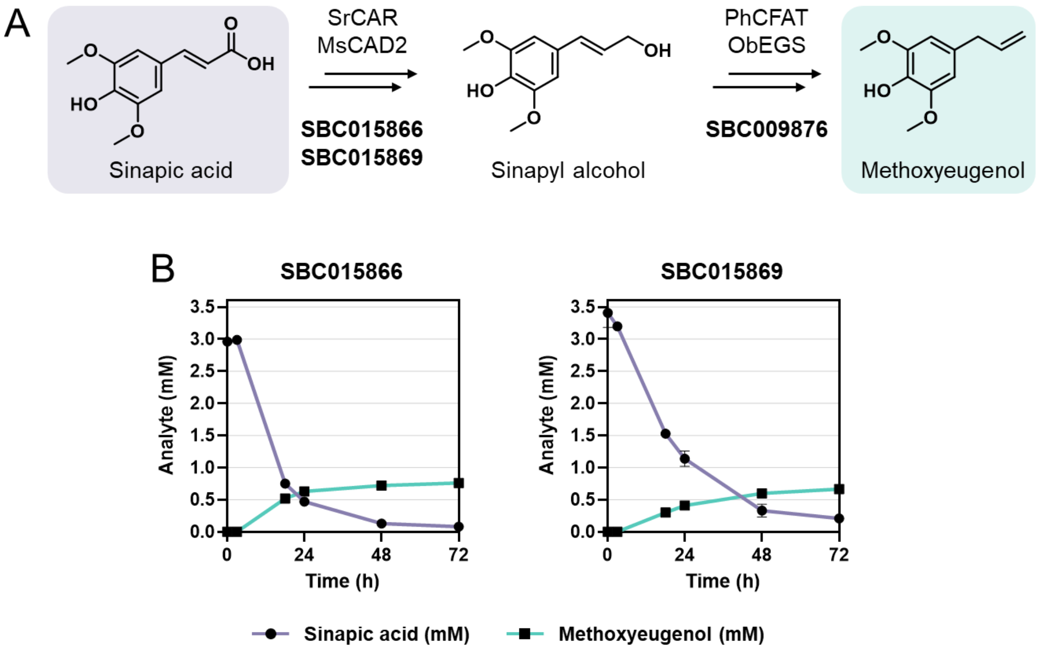

Figure S12. Bioconversion of sinapic acid into methoxyeugenol using the CAR-dependent pathway. **A** The methoxyeugenol biosynthesis pathway is split into two modules. The first module consists of SrCAR and MsCAD2 enzymes, catalysing the two-step reduction of sinapic acid, resulting in the formation of sinapyl alcohol. The second module comprises PhCFAT and ObEGS enzymes, catalysing the two-step conversion of sinapyl alcohol into methoxyeugenol. **B** Production of methoxyeugenol in *E. coli* NEB5α carrying SBC009876 in combination with one of the two indicated plasmids. At time point zero, cells were supplemented with 3 mM sinapic acid, and expression of pathway enzymes was induced by addition of IPTG. Error bars represent standard deviations of biological triplicates.

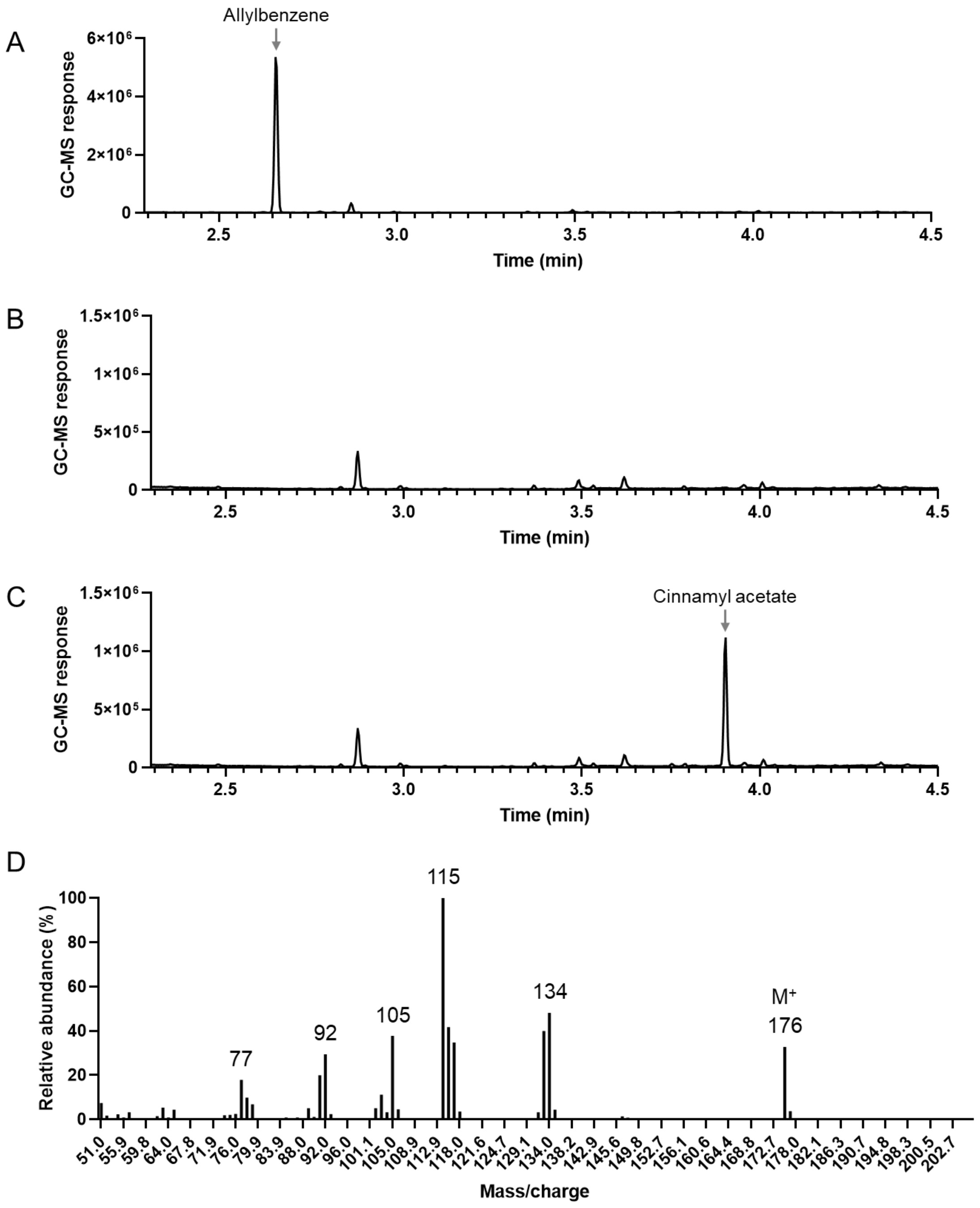

Figure S13. Bioconversion of cinnamic acid into cinnamyl acetate in *E. coli* NEB5α carrying SBC015866 and SBC009876. **A** The GC total ion chromatogram of 2,2,4-trimethylpentane (TMP) supplemented with *sec*-butylbenzene (internal standard) and allylbenzene. **B** The GC total ion chromatogram of TMP supplemented with *sec*-butylbenzene (negative control). **C** The GC total ion chromatogram of the TMP overlay from a culture of *E. coli* NEB5α carrying SBC015866 and SBC009876. Cells were supplemented with 3 mM cinnamic acid and expression of pathway enzymes was induced by addition of IPTG. Cultures were grown in headspace vials and samples were taken after 24 h. TMP was supplemented with *sec*-butylbenzene. **D** 70-eV electron ionisation mass spectrum of the prominent peak in the total ion chromatogram at 3.8 min shown in **C**. Dominant ions are labelled, as well as the molecular ion peak, M^+, of m/z 176.

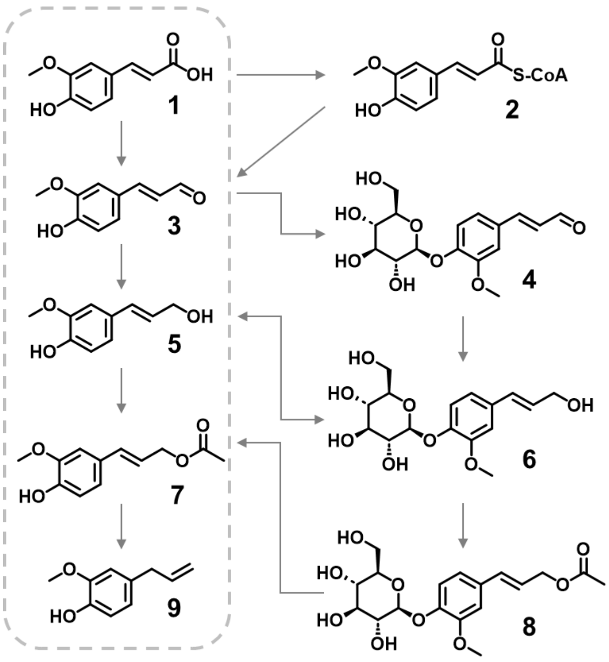

Pathway	Route
A	1 → 2 → 3 → 5 → 7 → 9
B	1 → 2 → 3 → 4 → 6 → 5 → 7 → 9
C	1 → 2 → 3 → 4 → 6 → 8 → 7 → 9
D	1 → 2 → 3 → 5 → 6 → 8 → 7 → 9
E	1 → 3 → 5 → 7 → 9

Figure S14. Top five retrosynthetic pathways for eugenol production from ferulic acid as substrate. Pathways A–D were the top hits from RetroPath2.0, while E was manually curated based on literature search. Compounds: 1, ferulic acid; 2, feruloyl-CoA; 3, coniferaldehyde; 4, coniferaldehyde glucoside; 5, coniferyl alcohol; 6, coniferyl alcohol beta-D-glucoside; 7, coniferyl acetate; 8, coniferyl acetate beta-D-glucoside; 9, eugenol.

References

[1] Robinson, C. J., Carbonell, P., Jervis, A. J., Yan, C., Hollywood, K. A., Dunstan, M. S., Currin, A., Swainston, N., Spiess, R., Taylor, S., Mulherin, P., Parker, S., Rowe, W., Matthews, N. E., Malone, K. J., Le Feuvre, R., Shapira, P., Barran, P., Turner, N. J., Micklefield, J., Breitling, R., Takano, E., and Scrutton, N. S. (2020) Rapid prototyping of microbial production strains for the biomanufacture of potential materials monomers, *Metab. Eng. 60*, 168-182.

[2] Lee, T. S., Krupa, R. A., Zhang, F., Hajimorad, M., Holtz, W. J., Prasad, N., Lee, S. K., and Keasling, J. D. (2011) BglBrick vectors and datasheets: a synthetic biology platform for gene expression, *J. Biol. Eng. 5*, 1-14.

[3] Jervis, A. J., Hanko, E. K., Dunstan, M. S., Robinson, C. J., Takano, E., and Scrutton, N. S. (2021) A plasmid toolset for CRISPR-mediated genome editing and CRISPRi gene regulation in *Escherichia coli*, *Microb. Biotechnol. 14*, 1120-1129.

[4] Klumbys, E., Zebec, Z., Weise, N. J., Turner, N. J., and Scrutton, N. S. (2018) Bio-derived production of cinnamyl alcohol *via* a three step biocatalytic cascade and metabolic engineering, *Green Chem. 20*, 658-663.
